# Novel Drosophila Viruses Encode Host-Specific Suppressors of RNAi

**DOI:** 10.1371/journal.ppat.1004256

**Published:** 2014-07-17

**Authors:** Joël T. van Mierlo, Gijs J. Overheul, Benjamin Obadia, Koen W. R. van Cleef, Claire L. Webster, Maria-Carla Saleh, Darren J. Obbard, Ronald P. van Rij

**Affiliations:** 1 Department of Medical Microbiology, Radboud University Nijmegen Medical Centre, Radboud Institute for Molecular Life Sciences, Nijmegen, The Netherlands; 2 Institut Pasteur, Viruses and RNA interference Unit and Centre National de la Recherche Scientifique, UMR 3569, Paris, France; 3 Institute of Evolutionary Biology and Centre for Immunity, Infection and Evolution, University of Edinburgh, Edinburgh, United Kingdom; Stanford University, United States of America

## Abstract

The ongoing conflict between viruses and their hosts can drive the co-evolution between host immune genes and viral suppressors of immunity. It has been suggested that an evolutionary ‘arms race’ may occur between rapidly evolving components of the antiviral RNAi pathway of *Drosophila* and viral genes that antagonize it. We have recently shown that viral protein 1 (VP1) of *Drosophila melanogaster* Nora virus (DmelNV) suppresses Argonaute-2 (AGO2)-mediated target RNA cleavage (slicer activity) to antagonize antiviral RNAi. Here we show that viral AGO2 antagonists of divergent Nora-like viruses can have host specific activities. We have identified novel Nora-like viruses in wild-caught populations of *D. immigrans* (DimmNV) and *D. subobscura* (DsubNV) that are 36% and 26% divergent from DmelNV at the amino acid level. We show that DimmNV and DsubNV VP1 are unable to suppress RNAi in *D. melanogaster* S2 cells, whereas DmelNV VP1 potently suppresses RNAi in this host species. Moreover, we show that the RNAi suppressor activity of DimmNV VP1 is restricted to its natural host species, *D. immigrans*. Specifically, we find that DimmNV VP1 interacts with *D. immigrans* AGO2, but not with *D. melanogaster* AGO2, and that it suppresses slicer activity in embryo lysates from *D. immigrans*, but not in lysates from *D. melanogaster*. This species-specific interaction is reflected in the ability of DimmNV VP1 to enhance RNA production by a recombinant Sindbis virus in a host-specific manner. Our results emphasize the importance of analyzing viral RNAi suppressor activity in the relevant host species. We suggest that rapid co-evolution between RNA viruses and their hosts may result in host species-specific activities of RNAi suppressor proteins, and therefore that viral RNAi suppressors could be host-specificity factors.

## Author Summary

Viruses and their hosts can engage in an evolutionary arms race. Viruses may select for hosts with more effective immune responses, whereas the immune response of the host may select for viruses that evade the immune system. These viral counter-defenses may in turn drive adaptations in host immune genes. A potential outcome of this perpetual cycle is that the interaction between virus and host becomes more specific. In insects, the host antiviral RNAi machinery exerts strong evolutionary pressure that has led to the evolution of viral proteins that can antagonize the RNAi response. We have identified novel viruses that infect different fruit fly species and we show that the RNAi suppressor proteins of these viruses can be specific to their host. Furthermore, we show that these proteins can enhance virus replication in a host-specific manner. These results are in line with the hypothesis that virus-host co-evolution shapes the genomes of both virus and host. Moreover, our results suggest that RNAi suppressor proteins have the potential to determine host specificity of viruses.

## Introduction

As obligate intracellular parasites, viruses modulate and exploit the host cellular environment for their replication. The host antiviral defense system restricts virus infections, and in turn, viruses dedicate a significant fraction of their coding capacity to produce factors that antagonize the antiviral immune response [1], [2]. Co-evolution of virus and host may therefore lead to a host-specific adaptation of viral counter-defense to the host antiviral defense system, which can contribute to host specificity of the virus [3].

The RNA interference (RNAi) pathway is a major antiviral defense system in plants, arthropods, nematodes and fungi [4]–[7] and has recently been suggested to control virus infection in mammals [8], [9]. Double stranded RNA (dsRNA), which is typically produced during virus infection but absent from non-infected cells [10], triggers the RNAi pathway. In insects, cleavage of viral dsRNA by the ribonuclease Dicer-2 (Dcr-2) generates viral small interfering RNAs (vsiRNAs) [11]–[23]. Dcr-2 and its binding partner R2D2 bind these vsiRNAs and load the small RNA duplexes into an Argonaute-2 (AGO2) containing RNA induced silencing complex (RISC) [24]. One strand of the vsiRNA is retained and guides the recognition and cleavage of complementary viral RNAs by AGO2 [11], [25]–[28]. In response, insect and plant viruses encode suppressors of RNAi (VSRs) to counteract the antiviral RNAi pathway [29]. Different mechanisms for RNAi suppression have been identified; for example, some VSRs bind long dsRNA and/or siRNAs to shield them from Dicer cleavage or prevent their loading into Argonaute [11], [30]–[38]. Other suppressors interact with Argonaute proteins to inhibit their activity or induce their degradation [14], [39]–[45].

The ongoing arms race with viruses can impose a strong selective pressure on immune genes of the host [46]. Consistent with this, *Dcr-2*, *R2D2*, and *AGO2* belong to the 3% fastest evolving genes in *D. melanogaster* and *D. simulans* and show very high rates of adaptive amino acid substitution with evidence for recent selective sweeps in multiple *Drosophila* species [47]–[49]. It has been hypothesized that this rapid adaptive evolution may be driven by antagonistic co-evolution with viral suppressors of RNAi [50], as the RNAi pathway continues to evolve new ways to escape viral antagonists, leading to counter-adaptations by viruses that require further adaptations in the RNAi pathway of the host. A potential outcome of this antagonistic co-evolution is that viral RNAi suppressors become specialized to suppress RNAi in their host species, while losing this activity in non-host species. This may be unlikely for viral antagonists that bind dsRNA, which often efficiently suppress RNAi in both host and non-host species, and in some cases even across kingdoms [51]–[55]. However, when viruses antagonize protein components of the RNAi pathway, there is ample opportunity for co-evolution and the evolution of host-specificity.

**Figure 1:**
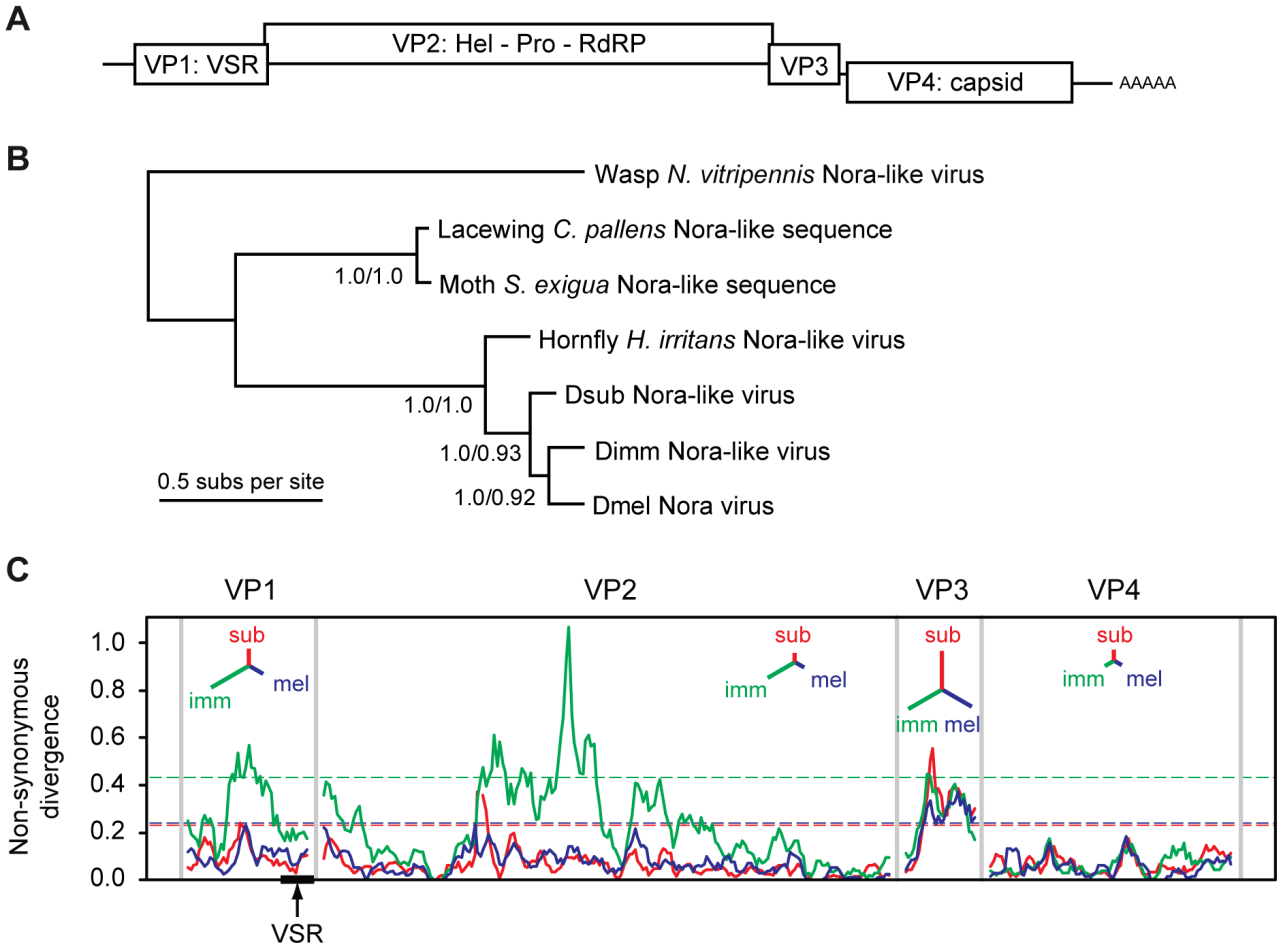
Phylogenetic analysis and non-synonymous divergence between Nora viruses. (**A**) Schematic representation of the genome organization of Nora virus. The virus encodes four open reading frames, some of which have a slight overlap. (**B**) Phylogenetic analysis of the most conserved Nora virus gene (VP4) suggests that the three Drosophila Nora-like viruses are each other's closest relatives, and that they are all closely related to the Nora-like sequence derived from *Haematobia irritans*. Although DimmNV appears to be most closely related to DmelNV based on VP4, the extreme divergence from the other Nora-like sequences may make the rooting unreliable. The tree presented is the mid-point rooted Bayesian maximum *a posteriori* tree (99% of the posterior set), the topology of which is identical to a Maximum Likelihood (ML) tree. Support values are given for internal nodes (Bayesian posteriors/ML bootstraps). The scale bar represents 0.5 amino acid substitutions per site. (**C**) A sliding-window analysis of nonsynonymous divergence between the three Drosophila Nora viruses, calculated as the number of nonsynonymous substitutions per nonsynonymous site. Dashed lines show a nominal 95% significance threshold for genome-wide peaks in divergence derived from randomisation tests, such that peaks crossing the lines are unlikely to occur by chance, given the overall divergence for that virus (colours correspond to the three viral lineages). Insets for each viral protein are unrooted trees with branch lengths proportional to overall divergence for that gene.

Nora virus of *Drosophila melanogaster* (DmelNV) is a recently identified natural fruit fly pathogen, which contains a single-stranded positive-sense RNA genome and appears to fall within the order of *Picornavirales* [56]. In contrast to other picorna-like viruses, DmelNV encodes four open reading frames: ORF 2 encodes replication proteins with clear homology to other *Picornavirales* members, ORF 4 encodes capsid proteins [57] (Figure 1A). No homology exists between the protein products of ORF1 or ORF3 and proteins of other viruses.

DmelNV causes persistent infections in laboratory stocks as well as in wild caught flies. Persistent infections are thought to reflect a dynamic equilibrium between host defense responses and viral counter-defense mechanisms [58]. The widespread abundance and persistent nature of DmelNV infections may suggest an equilibrium between antiviral RNAi and viral counter-defense, in which replication is restrained, but the infection is not cleared. Consistent with this, we recently showed that DmelNV is a target and a suppressor of the antiviral RNAi pathway [14]. We identified viral protein 1 (VP1), the product of open reading frame 1, as an RNAi suppressor that counteracts AGO2 mediated target RNA cleavage (slicer activity).

Here we present two novel Nora-like viruses identified by metagenomic sequencing of wild populations of *D. immigrans* (DimmNV) and *D. subobscura* (DsubNV), and we use these viral genomes to study RNAi antagonism from an evolutionary perspective. We find that the RNAi suppressor activity of DimmNV VP1 appears to be restricted to its natural host species, whereas DmelNV VP1 does not display any evidence of host specificity. We conclude that co-evolution between Nora viruses and their *Drosophila* hosts can result in host species-specific antagonism of AGO2, and therefore that viral suppressors of RNAi are candidate host specificity determinants.

## Results

### Identification of novel Nora-like viruses from *D. immigrans* and *D. subobscura*

RNAi genes evolve rapidly and adaptively in multiple species of *Drosophila* [47], [48]. We therefore hypothesized that the interaction between RNAi proteins and viral suppressors of RNAi may also evolve rapidly when viruses adapt to different hosts. In particular, optimization of such interactions in a specific host species may come at the cost of losing the interaction in non-host species. To test these hypotheses, we set out to identify novel Nora-like viruses from divergent *Drosophila* species.

During an exploratory RT-PCR survey of Nora virus prevalence in wild *Drosophila*, we identified two novel Nora-like viruses in wild populations of *D. immigrans* (DimmNV) and *D. subobscura* (DsubNV). Following this observation, we took a metagenomic RNA-sequencing approach to recover near-complete viral genomes for both viruses from population samples of *D. immigrans* and *D. subobscura* collected in the United Kingdom. The viral sequences were 12,265 nt and 12,276 nt, respectively (compare to 12,333 nt for DmelNV) and include all protein coding regions, a conserved CCTGGGSGGGGGTTA motif in their 5′ untranslated region, and a 3′ poly-A tract (Figure S1A). These novel viruses are more closely related to the Nora virus originally identified in *D. melanogaster* (DmelNV) [56] than they are to the Nora-like virus recently described in the horn fly *Haematobia irritans* [59], two Nora-like viruses identifiable in the transcriptomes of the lacewing *Chrysopa pallens* and the moth *Spodoptera exigua*, or the more distantly related Nora-like virus described in the wasp *Nasonia vitripennis* [60] (Figure 1B).

Overall, DmelNV is more divergent from DimmNV than it is from DsubNV (65% vs. 71% overall nucleotide identity, respectively), but phylogenetic analysis based on the coat protein (VP4) suggests that DmelNV and DimmNV may be each other's closest relatives. The low genome-wide nonsynonymous to synonymous substitution ratio (dN/dS = 0.076, SE = 0.003) estimated by PAML [61] indicates that evolution of the protein sequence is highly constrained. However, divergence between the three viruses is too high to reliably estimate dS [62], [63] and the estimated dN/dS may represent an upper limit.

Amino-acid divergence between the viruses varies substantially between genes (Figure 1C). For example, amino-acid identity between DimmNV and DmelNV varies from 82% for VP4 (capsid) to only 43% for VP3 (unknown function), with VP1 showing an intermediate level of conservation (51% amino acid identity). A sliding-window analysis of nonsynonymous divergence shows that DimmNV is much more divergent from DmelNV and DsubNV in VP1 and VP2, but that the three viruses are equidistant from each other in VP3 and VP4. This may be a result of host-mediated selection, perhaps reflecting the closer relationship between *D. melanogaster* and *D. subobscura*, or it may be a result of recombination in the history of these three viruses.

### VP1 of Dimm and Dsub Nora-like viruses do not suppress RNAi in *D. melanogaster* S2 cells

**Figure 2:**
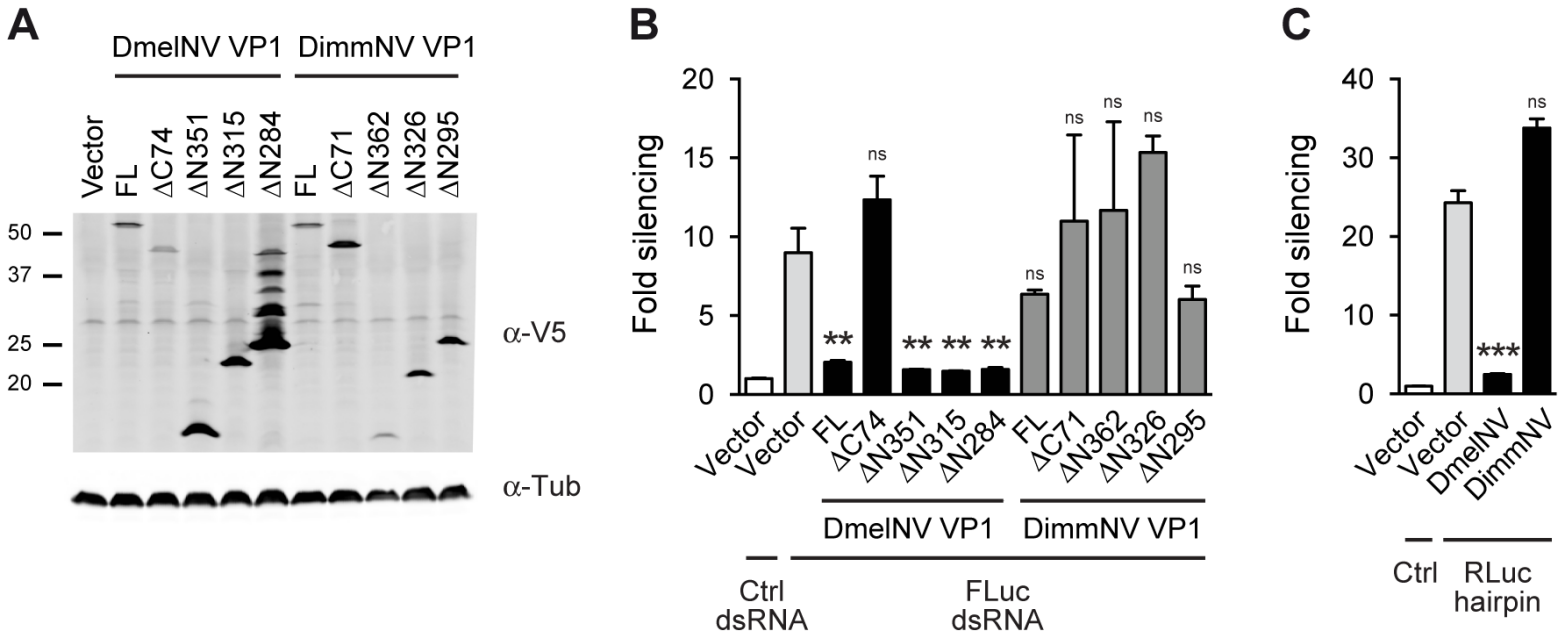
VP1 suppressor activity is species-specific. (**A**) Western blot analysis of S2 cells expressing V5 epitope-tagged VP1 from *D. melanogaster* Nora virus (DmelNV) and *D. immigrans* Nora-like virus (DimmNV). S2 cells were transfected with plasmids encoding full-length VP1 (FL) and C-terminal (ΔC) or N-terminal (ΔN) deletions thereof. Expression of the VP1 constructs was analyzed by western blot using an anti-V5 (α-V5) antibody. Detection of tubulin with anti-tubulin (α-tub) antibody was used as a loading control. Molecular mass (in kDa) is indicated on the left. For DmelNV VP1^ΔN284^, bands of lower mobility were observed in addition to the expected 26 kDa protein, the nature of which remains unknown. Note that these additional bands are not consistently observed (Figure S2A, lane 5, and [14]). (**B**) RNAi sensor assay in S2 cells. Firefly luciferase (Fluc) and *Renilla* luciferase (Rluc) reporter plasmids were transfected into S2 cells, together with plasmids encoding the indicated VP1 constructs. Two days after transfection, S2 cells were soaked in either control (Ctrl) dsRNA or Fluc dsRNA, and luciferase activities were measured the next day. Fluc counts were normalized to Rluc counts, and presented as fold silencing relative to the corresponding control dsRNA treatment. (**C**) Hairpin-based RNAi sensor assay in S2 cells. S2 cells were transfected with plasmids coding for Fluc, Rluc, and an Rluc-hairpin RNA together with a control vector (Vector) or plasmids encoding the N-terminal deletion mutants of DmelNV VP1^ΔN284^ or DimmNV VP1^ΔN295^. Rluc counts were normalized to Fluc counts, and presented as fold silencing over non-hairpin control transfections. Bars in Panels B and C represent means and standard deviations of three independent biological replicates. One-way ANOVA followed by Dunnett's *post hoc* test was used to evaluate whether VP1 constructs significantly suppressed RNAi relative to the vector control (light gray bar). ** *P*<0.01; *** *P*<0.001; ns, not significant.

To test whether the interaction between antiviral RNAi components and viral RNAi antagonists is host specific, we first analyzed whether the DimmNV and DsubNV VP1 proteins are able to suppress RNAi in the S2 cell line from *D. melanogaster*. To this end, we cloned the full-length (FL) VP1 sequences and N- and C-terminal deletion mutants thereof (ΔN and ΔC) as N-terminal fusions to the V5 epitope in an insect expression plasmid (Figure S1B). We verified expression of the DimmNV VP1 constructs by western blot after transfection in *Drosophila* S2 cells (Figure 2A). With the exception of the DimmNV VP1^ΔN362^, all DimmNV VP1 constructs were expressed at least at the level of DmelNV VP1^FL^ that efficiently suppresses RNAi in reporter assays in S2 cells [14]. We then analyzed the ability of the DimmNV VP1 constructs to suppress RNAi in reporter assays. We transfected S2 cells with firefly and *Renilla* luciferase (Fluc and Rluc) reporter plasmids along with VP1 expression plasmids, and induced silencing of the Fluc reporter by soaking the cells in Fluc specific dsRNA. As reported earlier [14], all DmelNV VP1 constructs, except DmelNV VP1^ΔC74^, suppressed RNAi-mediated silencing of the Fluc reporter. In contrast, none of the DimmNV VP1 constructs efficiently suppressed silencing of the reporter (Figure 2B). To confirm these results, we used an RNAi sensor assay that is independent of dsRNA uptake by S2 cells. In this sensor assay, the Rluc reporter is silenced by expression of an inverted repeat that folds into an Rluc-specific RNA hairpin. In line with the previous RNAi sensor assay, DimmNV VP1 did not suppress hairpin-induced silencing of the Rluc reporter in *D. melanogaster* S2 cells, whereas DmelNV VP1 efficiently suppressed RNAi (Figure 2C). In addition, we tested if the VP1 constructs can suppress RNAi in a sensor assay in which silencing is induced by co-transfection of siRNAs. Also in this assay, DimmNV VP1 was unable to suppress silencing of the Fluc reporter, whereas DmelNV VP1 efficiently suppressed RNAi-based silencing (Figure S2). Similarly, the DsubNV VP1 constructs were unable to suppress long dsRNA or siRNA induced RNAi in *D. melanogaster* derived S2 cells (Figure S2A–C). Moreover, recombinant DmelNV VP1 efficiently suppressed AGO2 slicer activity in embryo lysates of *D. melanogaster*, whereas DsubNV VP1 was unable to do so (Figure S2D). Together, these results indicate that VP1 of DimmNV and DsubNV do not suppress RNAi in *D. melanogaster*.

### DimmNV VP1 inhibits slicer activity in its natural host species, *D. immigrans*

The inability of DimmNV VP1 and DsubNV VP1 to suppress RNAi in *Drosophila* S2 cells may be explained in two ways. First, viral RNAi suppressors may have a species-specific activity, following the prediction that prolonged virus-host coevolution may result in efficient RNAi suppressive activity in host species but not in non-host species. Second, some Nora-like viruses may either be unable to suppress RNAi, or they may encode RNAi suppressor activity in different regions of the viral genome, as has been observed for members of a single plant virus family [64]–[66]. To address the first possibility, we tested the ability of DimmNV VP1 and DmelNV VP1 to suppress RNAi in both host species using *in vitro* RNA cleavage (slicer) assays [67] in lysates of embryos from *D. melanogaster* and *D. immigrans*. Unfortunately, we were not successful in producing slicer competent lysates for *D. subobscura*. Moreover, members of the *Drosophila* obscura group encode multiple AGO2-like proteins of unknown function [68]. These proteins may be functionally redundant, and may not all be targeted by a VSR. We therefore chose not to include *D. subobscura* and DsubNV in subsequent analyses.

**Figure 3:**
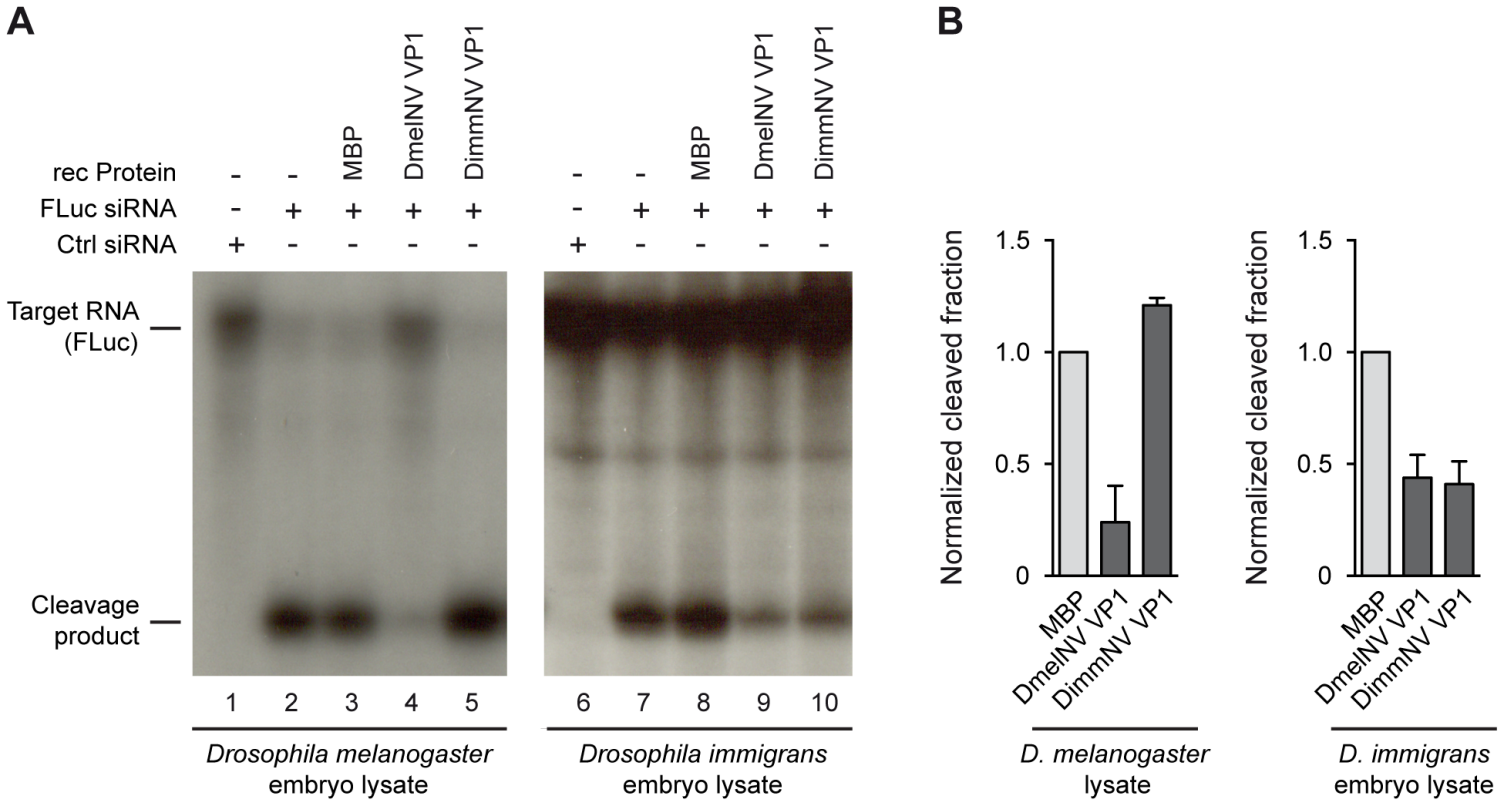
Species-specific inhibition of AGO2 slicer activity. (**A**) *In vitro* RNA cleavage (slicer) assays in lysates from *D. melanogaster* embryos (left panel) or *D. immigrans* embryos (right panel). Radioactively cap-labelled target RNA was incubated in embryo lysate together with a non-specific control siRNA (lanes 1 and 6) or a target specific siRNA (lanes 2–5, 7–10). Target cleavage was determined either in the absence of recombinant protein (lanes 2 and 7) or in the presence of 0.3 µM of MBP (lanes 3 and 8), MBP-DmelNV VP1 (lanes 4 and 9), or DimmNV VP1 (lanes 5 and 10). (**B**) Quantification of target cleavage in *D. melanogaster* and *D. immigrans* embryo lysate in the presence of MBP, DmelNV VP1, or DimmNV VP1 protein. The fraction of cleaved RNA was determined by dividing the intensity of the cleavage product by the total intensity of cleavage product and non-cleaved target. Data are normalized to MBP. Bars represent means and standard deviations of two independent experiments.

In slicer assays, RNAi dependent cleavage of a ^32^P cap-labelled target RNA is induced by the addition of a target specific siRNA. Since the target RNA is radio-labelled at its 5′ cap, the 5′ cleavage product can be visualized by autoradiography after polyacrylamide gel electrophoresis. As expected, in both *D. melanogaster* and *D. immigrans* embryo lysates a specific cleavage product was observed after incubation with a target specific siRNA (Figure 3A, lanes 2 and 7). In line with our earlier report [14], recombinant DmelNV VP1 protein potently inhibited cleavage of the target RNA in *D. melanogaster* embryo lysate, whereas the control, Maltose Binding Protein (MBP), was unable to do so (Figure 3A, compare lanes 3 and 4). In contrast, recombinant DimmNV VP1 protein did not inhibit slicer activity in *D. melanogaster* embryo lysate (Figure 3A, lane 5), which is in line with our observation that DimmNV VP1 did not suppress RNAi in cell-based reporter assays in *D. melanogaster* cells (Figure 2). Surprisingly, in the *D. immigrans* embryo lysate both the DmelNV VP1 and the DimmNV VP1 protein substantially inhibited target RNA cleavage (Figure 3A, lanes 9 and 10). Again, as expected, the MBP control protein did not inhibit slicer activity (Figure 3A, lane 8). Quantification of independent experiments indicates that both DmelNV and DimmNV VP1 proteins suppressed slicer activity to a similar extent in the *D. immigrans* embryo lysate (Figure 3B). These results, together with those from the cell-based reporter assays, indicate that DimmNV VP1 inhibits slicer activity in its natural host *D. immigrans*, but is unable to suppress RNAi in a heterologous *D. melanogaster* background. In contrast, DmelNV VP1 inhibits slicer activity in both a *D. melanogaster* and a *D. immigrans* background.

### DmelNV VP1 interacts with Dmel AGO2

**Figure 4:**
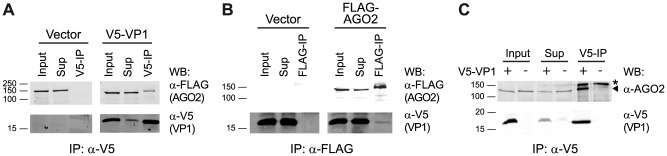
DmelNV VP1 interacts with Dmel AGO2 in S2 cells. (**A**) Western blot (WB) analysis of V5 immunoprecipitation on lysates from S2 cells transfected with a FLAG-AGO2 expression plasmid and either V5-tagged DmelNV VP1 (V5-VP1) or V5-control plasmid (Vector). The epitope-tagged proteins were detected in the input, supernatant after immunoprecipitation (Sup), and the immunoprecipitate (V5-IP) with the indicated antibodies. (**B**) FLAG immunoprecipitation of lysates from S2 cells transfected with V5-tagged DmelNV VP1 (V5-VP1) and either FLAG-AGO2 or FLAG-control plasmids (Vector), followed by western blot analysis with the indicated antibodies. (**C**) V5 immunoprecipitation of lysates from S2 cells transfected with V5-tagged DmelNV VP1 (+) or V5-control (−) plasmids. After SDS-PAGE, endogenous AGO2 or DmelNV VP1 proteins were detected by western blot using anti-AGO2 (α-AGO2) and anti-V5 (α-V5) antibody, respectively. Asterisk (*) indicates a non-specific background band; triangle indicates AGO2. The DmelNV VP1^ΔN351^ construct was used in these experiments.

We recently showed that DmelNV VP1 inhibits RNA cleavage (slicer) activity of a pre-assembled RISC in *D. melanogaster* [14], suggesting that VP1 interacts with AGO2 to suppress its catalytic activity. To investigate a physical interaction between VP1 and AGO2, we analyzed DmelNV VP1 immunoprecipitations (IPs) for the presence of AGO2. To this end, we transfected S2 cells with a functional V5 epitope-tagged VP1 construct (V5-VP1) that encodes the C-terminal 124 amino acids of VP1 along with a FLAG-tagged AGO2 cDNA construct. Immunoprecipitation of V5-VP1 resulted in specific co-precipitation of the FLAG-AGO2 protein (Figure 4A). In contrast, the vector control failed to co-purify FLAG-AGO2. To confirm the interaction between VP1 and AGO2, we performed the reverse experiment. IP of FLAG-AGO2 protein co-precipitated V5-VP1, while a FLAG-control vector was unable to do so (Figure 4B). Although the interaction between VP1 and AGO2 is evident, only a minor fraction of VP1 was immunoprecipitated along with AGO2. This observation is in agreement with our microscopic analyses, in which only a small fraction of FLAG-AGO2 protein co-localizes with VP1-EGFP (data not shown). To confirm these results, we immunoprecipitated V5-VP1 protein and probed for endogenous AGO2 in the IP fraction. As expected, we observed a strong enrichment of endogenous AGO2 protein after VP1 IP, whereas IP of cells transfected with control plasmid did not co-precipitate AGO2 protein (Figure 4C). These results indicate that DmelNV VP1 interacts with Dmel AGO2 in *Drosophila* S2 cells.

### Species-specific interaction between DimmNV VP1 and Dimm AGO2

**Figure 5:**
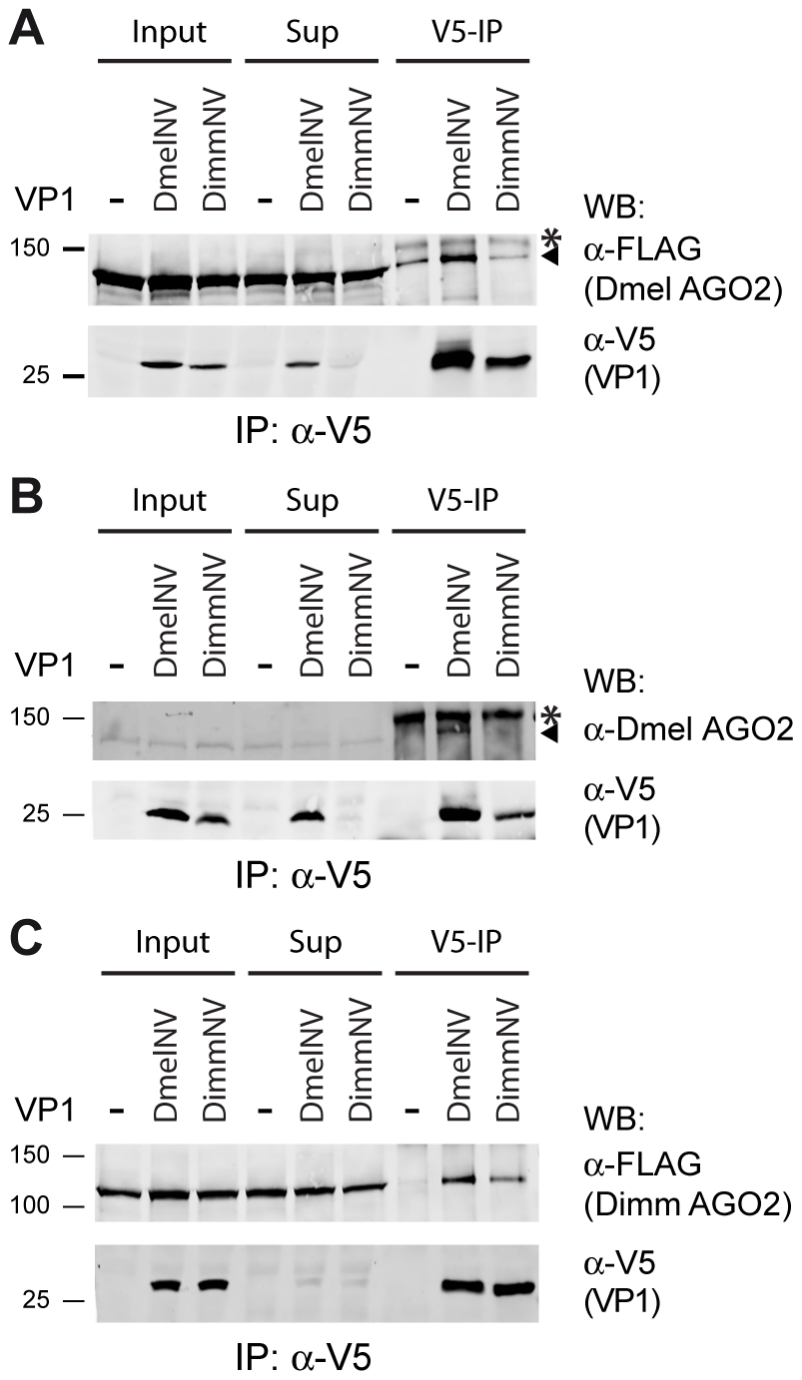
Species-specific interaction between VP1 and AGO2. (**A**) V5 Immunoprecipitation (V5-IP) of lysates from S2 cells transfected with FLAG-tagged Dmel AGO2 expression plasmid and either V5-tagged DmelNV VP1, DimmNV VP1, or V5-control plasmids (−). Input, supernatant after immunoprecipitation (Sup), and the immunoprecipitate (V5-IP) were analyzed by western blot (WB) using anti-V5 (α-V5) or anti-FLAG (α-FLAG) antibodies. (**B**) V5 immunoprecipitation of S2 cells transfected with plasmids encoding V5-tagged DmelNV VP1, DimmNV VP1, or V5-control vector (−). Input, sup, and IP fractions were analyzed by western blot using antibodies for endogenous AGO2 (α-Dmel AGO2) and V5 (α-V5). (**C**) V5 immunoprecipitation on lysates from S2 cells co-transfected with plasmids encoding FLAG-tagged Dimm AGO2 and either V5-tagged DmelNV VP1, DimmNV VP1, or V5-control vector (−). VP1 and Dimm AGO2 proteins were detected on western blot using anti-V5 (α-V5) and anti-FLAG (α-FLAG) antibodies, respectively. Asterisks (*) indicate a non-specific background band; triangles indicate AGO2. For these experiments the corresponding DmelNV VP1^ΔN284^ and DimmNV VP1^ΔN295^ constructs were used (Figure S1).

These data and the results from our previous report [14] indicate that DmelNV VP1 interacts with Dmel AGO2 to antagonize the antiviral RNAi response. Similarly, given the observation that DimmNV VP1 suppresses slicer activity in *D. immigrans* lysates, it is likely that DimmNV VP1 interacts with Dimm AGO2. We hypothesized that the inability of DimmNV VP1 to suppress RNAi in *D. melanogaster* may then be due to an inefficient interaction with Dmel AGO2. To test these hypotheses, we analyzed VP1 interactions with host and non-host AGO2 proteins by co-IPs. First, we co-expressed V5 epitope-tagged DmelNV VP1 or DimmNV VP1 with Dmel FLAG-AGO2 in S2 cells and immunopurified the VP1 proteins using V5 affinity beads. As controls, we analyzed IPs of cells transfected with empty vector. As observed above (Figure 4), IP of DmelNV VP1 co-precipitated Dmel FLAG-AGO2 protein. In contrast, IP of DimmNV VP1 did not enrich Dmel FLAG-AGO2 in the IP fraction, compared to IP of the vector control (Figure 5A). To confirm these results, we analyzed the interaction between VP1 proteins and endogenous *D. melanogaster* AGO2. While DmelNV VP1, but not the control vector, co-precipitated endogenous Dmel AGO2 (Figure 4C, Figure 5B), DimmNV VP1 failed to co-IP endogenous Dmel AGO2, which mirrors our observation with epitope-tagged Dmel AGO2. These observations imply that the inability of DimmNV VP1 to suppress RNAi in *D. melanogaster* is due to its inability to efficiently interact with Dmel AGO2.

We next set out to analyze the interaction of DimmNV VP1 with Dimm AGO2. To this end, we cloned the *D. immigrans* AGO2 cDNA sequence downstream of the FLAG epitope (Dimm FLAG-AGO2). As expected, the predicted protein domains of Dimm FLAG-AGO2 are similar to those of Dmel AGO2, suggesting that the overall protein structure of Dimm and Dmel AGO2 are alike. Overall amino acid identity is 56% (63% when excluding the poly-glutamine repeats), with a higher level of conservation in the PIWI domain (77% identity) than in the PAZ domain (45% identity). We thus analyzed the interaction of DmelNV VP1 or the DimmNV VP1 with Dimm FLAG-AGO2 in co-IP. Both DmelNV VP1 and DimmNV VP1 efficiently co-purified the Dimm AGO2 protein (Figure 5C). These results show that AGO2-VP1 interactions correlate with RNAi suppressor activity: DmelNV VP1 interacts with both Dmel and Dimm AGO2 and suppresses slicer activity of these hosts; DimmNV VP1 interacts with Dimm AGO2, but not Dmel AGO2, and suppresses slicer activity in *D. immigrans*, but not in *D. melanogaster*.

### DimmNV VP1 specifically suppresses Dimm AGO2 activity

The species-specific interaction of DimmNV VP1 with Dimm AGO2 suggests that this interaction is the major determinant for the observed species specificity in slicer activity. To test this hypothesis, we set out to reconstitute Dimm AGO2-based silencing in *D. melanogaster* S2 cells and to analyze whether DimmNV VP1 could suppress this reconstituted pathway. To this end, we reduced endogenous AGO2 expression in *D. melanogaster* S2 cells using RNAi, and rescued its activity with either a Dmel AGO2 or Dimm AGO2 cDNA construct.

**Figure 6:**
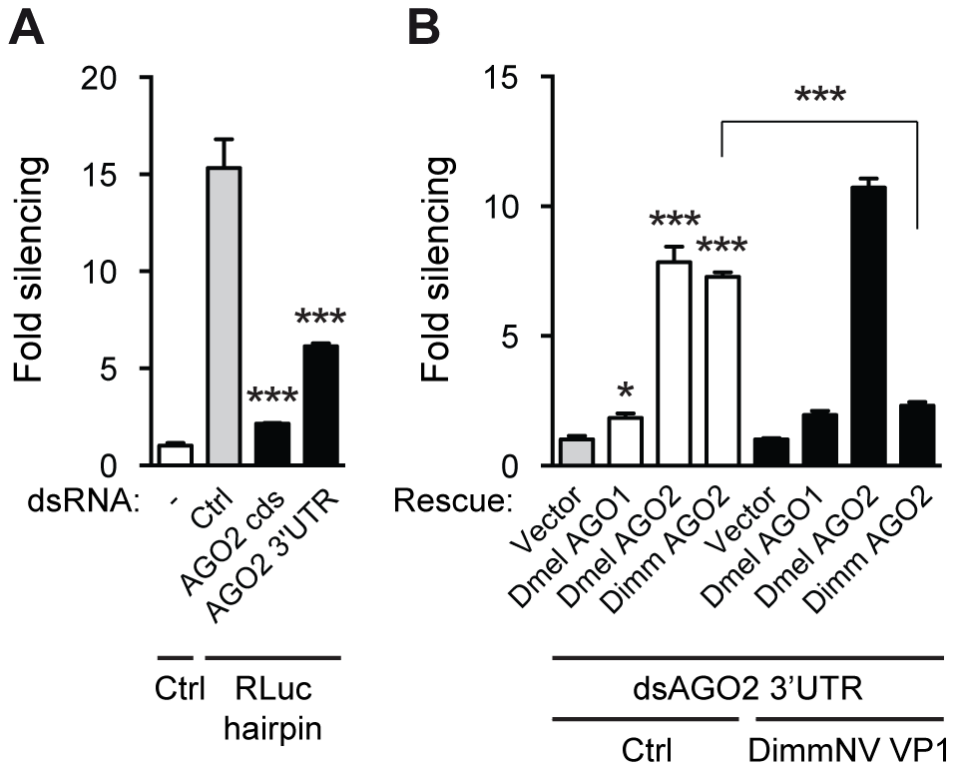
DimmNV VP1 inhibits Dimm AGO2 function. (**A**) RNAi reporter assay based on hairpin-induced silencing of an Rluc reporter. The experiment was performed as described in the legend to Figure 2C, except that a non-specific control dsRNA (Ctrl) or dsRNA targeting the coding sequence or the 3′UTR of Dmel AGO2 (AGO2 CDS and AGO2 3′UTR, respectively) was co-transfected along with the reporter plasmids. Bars represent means and standard deviations of three biological replicates. One-way ANOVA followed by Dunnett's *post hoc* test was used to evaluate loss of silencing by AGO2 dsRNA compared to control dsRNA treated samples (light gray bars). (**B**) Rescue of endogenous AGO2 knockdown by *D. immigrans* AGO2 and suppression thereof by DimmNV VP1. Endogenous AGO2 expression was reduced by dsRNA targeting the AGO2 3′UTR, which was transfected along with luciferase reporter plasmids, Rluc hairpin plasmid, and control plasmid (Vector) or expression plasmids encoding *D. melanogaster* AGO1 (Dmel AGO1), AGO2 (Dmel AGO2), or *D. immigrans* AGO2 (Dimm AGO2). Control vector (Ctrl, white bars) or a plasmid encoding *D. immigrans* Nora virus VP1 (DimmNV VP1^ΔN295^, black bars) was co-transfected to analyze the ability of DimmNV VP1 to suppress Dimm and Dmel AGO2-mediated silencing. Data are presented as fold silencing relative to the corresponding vector control transfection. Bars represent means and standard deviations of three biological replicates. One-way ANOVA followed by Dunnett's *post hoc* test was used to evaluate whether AGO expression rescued silencing relative to the vector control in the absence of VP1 (light gray bar). A Student's T-test was used to analyze whether loss of silencing by expression of DimmNV VP1 was significant. * *P*<0.05; *** *P*<0.001.

First, we assessed the efficacy of knockdown of AGO2 expression in S2 cells using dsRNA targeting the coding sequence (CDS) or the 3′ untranslated region (3′ UTR) of the endogenous Dmel AGO2 transcript. To monitor AGO2 activity in these S2 cells we induced RNAi with the Rluc-specific RNA hairpin (described in Figure 2C). Compared to a non-specific dsRNA control, dsRNA against the CDS or the 3′UTR of AGO2 efficiently reduced hairpin-induced silencing of the Rluc reporter (Figure 6A). This experiment thus creates the opportunity to knock down endogenous AGO2 expression with UTR-targeting dsRNA and rescue silencing defects with Dmel AGO2 or Dimm AGO2 cDNA constructs that lack the AGO2 3′UTR sequence and are therefore not targeted by this RNAi approach. Strikingly, both Dmel AGO2 and Dimm AGO2 rescued silencing activity in *D. melanogaster* cells, whereas Dmel AGO1 only slightly increased silencing activity relative to the vector control (Figure 6B). These results indicate that Dimm AGO2 is fully functional in a *D. melanogaster* background and that the limited sequence identity to Dmel AGO2 does not impede its ability to interact with Dmel Dcr-2, R2D2 and other components of the *D. melanogaster* RISC complex.

Using this AGO2 rescue assay, we investigated whether DimmNV VP1 suppressed Dmel and Dimm AGO2-mediated silencing. DimmNV VP1 expression did not impede Dmel Ago2-mediated RNAi (Figure 6B), which is in line with our observations that DimmNV VP1 did not inhibit RNAi in *D. melanogaster* S2 cells (Figure 2A). In contrast, we observed that Dimm AGO2-mediated silencing was efficiently suppressed by DimmNV VP1 (Figure 6B). We were unable to analyze DmelNV VP1 in this assay, as its potent RNAi suppressive activity would impede silencing of endogenous Dmel AGO2, which is required for this assay.

Together, these results indicate that the interaction of VP1 with AGO2 is the major determinant for its RNAi suppressive activity. Moreover, these data imply that the VP1-AGO2 interaction is a major determinant for the species-specific effects of VP1.

### DmelNV and DimmNV VP1 enhance viral RNA production of recombinant Sindbis virus in a host species-specific manner

**Figure 7:**
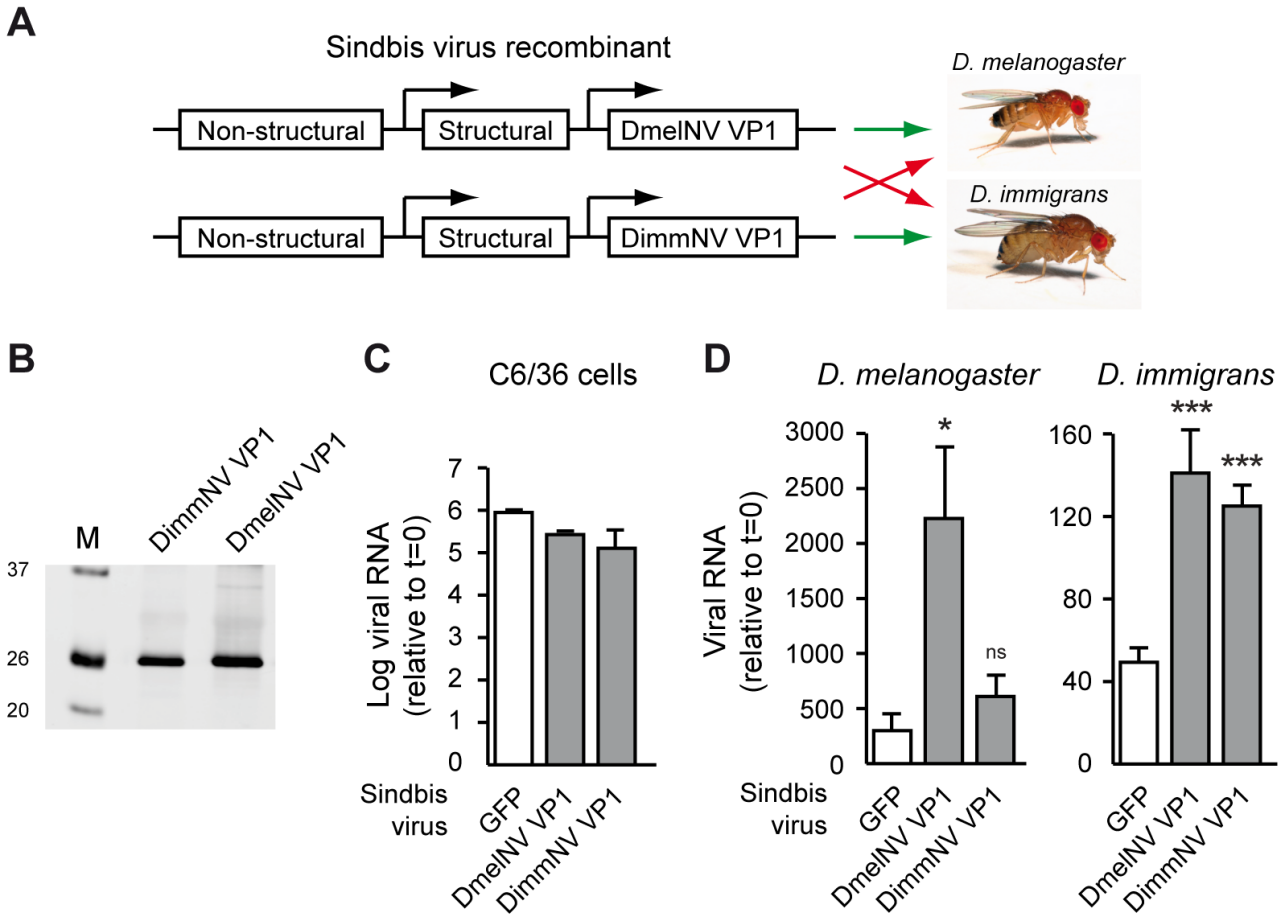
Viral RNAi suppressors are host species-specific pathogenicity determinants. (**A**) Outline of the experimental set-up. VP1 from DimmNV and DmelNV was expressed under control of a duplicated subgenomic promoter in Sindbis virus and recombinant viruses were tested for replication in two *Drosophila* host species. A GFP-expressing Sindbis virus recombinant was included as a control. (**B**) Western blot analysis of BHK cells infected with Sindbis recombinants expressing the indicated transgenes. Expression of the VP1 constructs was analyzed by western blot using anti-V5 antibody. M, size marker; molecular mass (in kDa) is indicated on the left. (**C**) Viral RNA production of recombinant Sindbis viruses in *Dicer-2* deficient cells. C6/36 cells were inoculated with the indicated Sindbis recombinants (multiplicity of infection of 0.01) and viral RNA levels were analyzed at 24 h post inoculation by qRT-PCR. Data are normalized to viral RNA levels in cells harvested directly after inoculation (t = 0). Bars indicate means and SEM (n = 3 biological replicates). (**D**) Viral RNA production in *D. melanogaster* (left panel) and *D. immigrans* (right panel) infected with recombinant Sindbis viruses expressing the indicated VP1 transgenes or, as a control, GFP. Viral RNA levels were measured at 7 days post inoculation (dpi) by qRT-PCR and normalized to viral RNA levels in flies that were harvested immediately after inoculation (t = 0). Mean viral RNA levels and SEM (n = 3 biological replicates) are shown. A Student's T-test was used for pairwise comparison between the Sindbis VP1 recombinants and Sindbis-GFP (white bars). * *P*<0.05; *** *P*<0.001; ns, not significant.

Together, our data suggest that the interaction between viral RNAi suppressors and its cellular protein targets can be host specific. Thus, DimmNV VP1 suppresses AGO2-mediated silencing of its *D. immigrans* host, but not in non-host *D. melanogaster*; in contrast, DmelNV VP1 seems to be more promiscuous and inhibits AGO2-mediated RNAi in both *D. melanogaster* and *D. immigrans*. An exciting hypothesis is therefore that the species-specific interaction between VP1 and AGO2 can mediate host specificity of *Drosophila* Nora viruses. To test this hypothesis, we generated replication-competent Sindbis virus (SINV) recombinants expressing either DimmNV VP1, DmelNV VP1, or, as a control, GFP from a second subgenomic promoter (Figure 7A). As SINV is restricted by antiviral RNAi in *Drosophila* [14], [69], suppression of RNAi by expression of an exogenous viral RNAi suppressor is expected to yield higher viral RNA levels. Indeed, we previously showed that a DmelNV VP1 transgene renders SINV more pathogenic in *D. melanogaster* in an RNAi-dependent manner [14]. Our hypothesis thus predicts that the DimmNV VP1-expressing SINV recombinant reaches higher viral RNA levels than Sindbis-GFP in *D. immigrans*, but not in *D. melanogaster*, whereas Sindbis-DmelNV VP1 is expected to produce more viral RNA than SINV-GFP in both *D. immigrans* and *D. melanogaster*. We first verified stable expression of the VP1 transgenes by SINV recombinants by western blot (Figure 7B). Next, we analyzed whether SINV recombinants are equally replication competent in the C6/36 cell line that does not express functional Dicer-2. In this background, the presence of the VP1 transgene should not provide a replicative advantage over the GFP transgene. Indeed, VP1-expressing Sindbis virus recombinants replicated to slightly lower viral RNA levels than SINV-GFP in C6/36 cells (Figure 7C), indicating that none of the recombinant viruses suffer from major replication defects.

We next analyzed replication of SINV recombinants in *D. melanogaster* and *D. immigrans* hosts. As expected [14], in *D. melanogaster* the DmelNV VP1 transgene strongly increased viral RNA levels compared to SINV-GFP infection at 7 days post-infection (dpi) (Figure 7D, left panel). In general, *D. immigrans* only supported low levels of SINV replication. Nevertheless, in this host DmelNV VP1 increased SINV RNA levels, which is in line with our observation that this protein has RNAi suppressive activity in both hosts. The effects of DimmNV on viral RNA production also mirrored host specificity of its biochemical activity. Viral RNA levels of SINV-DimmNV VP1 were similar to SINV-GFP RNA levels in *D. melanogaster* (Figure 7D, left panel). In *D. immigrans* however, a strong increase in viral RNA levels was observed. Thus, DimmNV VP1 enhances viral RNA levels of recombinant Sindbis virus in a host species-specific manner, suggesting that the interaction of viral RNAi suppressors with AGO2 may be a determinant of host-specific pathogenicity.

## Discussion

Viruses and their hosts engage in an ongoing arms race in which viral counter-defense mechanisms drive the adaptive evolution of host immune genes, which in turn requires ongoing counter-adaptations in viral immune antagonists [3], [46]. This cycle of adaptation and counter-adaptation may result in species-specific interactions between virus and host [46], [70].

The antiviral RNAi genes *R2D2, Dcr-2* and *AGO2* belong to the 3% fastest evolving genes of *Drosophila melanogaster* and show evidence of positive selection in multiple species [47]. Strikingly, rapid evolution is observed in the antiviral RNAi pathway, whereas the microRNA pathway does not show evidence for rapid evolution. It is therefore possible that antagonistic host-parasite interactions – either through prolonged coevolution or through invasion by novel pathogens – are responsible for the observed rapid adaptive evolution in RNAi genes. Similarly, reciprocal antagonism between microbial pathogens and their hosts has been suggested to be the cause of positive selection observed in other insect immune genes, such as Relish and α-2-Macroglobulin [71]–[73].

Nora virus is a positive-sense RNA virus that was recently identified in laboratory stocks of *Drosophila melanogaster* [56]. Its unique genome organization and capsid structure suggests that Nora virus is the founding member of a novel virus family [57]. We report here that divergent Nora-like virus sequences are found in wild-caught *D. immigrans* and *D. subobscura* flies. Together with the recent isolation of Nora-like virus sequences from the horn fly *Haematobia irritans* and the parasitoid wasps *Nasonia vitripennis* and *N. giraulti* [59], [60] and the presence of Nora-like sequences in the transcriptomes of the lacewing *Chrysopa pallens* and the moth *Spodoptera exigua* (this report), our observations suggest that Nora virus is a member of a large family of widespread pathogens that infects multiple insect species.

Although little is known regarding the natural host range of Nora viruses, it is worth noting that neither of our population samples of *D. immigrans* or *D. subobscura* contained sequences derived from the other Nora lineages (i.e. DmelNV was not identified in *D. immigrans* or *D. subobscura*, and similarly for the other Nora-like viruses [DJO, unpublished data]), despite being initially collected as mixed samples of multiple *Drosophila* species. It is therefore possible that, as is the case for the purely vertically transmitted Sigma viruses, Nora viruses rarely move between hosts [74].

Plant and insect viruses can suppress the antiviral RNAi pathway of their hosts via a variety of mechanisms [11], [14], [29], [30], [35], [38], [43], [75]. We recently showed that Nora virus VP1 suppresses RNAi by inhibiting AGO2 slicer activity of a pre-assembled RISC [14]. Here we show that the RNAi suppressor activity of VP1 from Nora-like viruses can be host specific and that its RNAi suppressive activity correlates with its ability to interact with AGO2. DimmNV VP1 efficiently interacts with Dimm AGO2 and suppresses AGO2-mediated slicer activity in *D. immigrans* embryo lysates. In contrast, DimmNV VP1 was unable to suppress RNAi in *D. melanogaster* cells, did not interact with Dmel AGO2, and did not inhibit slicer activity in *D. melanogaster* embryo lysates. These results are consistent with a model in which adaption and co-evolution of DimmNV with its host resulted in a species-specific AGO2-VP1 interaction.

Our findings have important practical implications. Experimentally amenable model systems, such as *Drosophila melanogaster* or *Arabidopsis thaliana*, are often used to identify and characterize viral suppressors of RNAi, including those of viruses that naturally do not infect these hosts. Our observation that RNAi suppressor proteins may have species-specific activity suggests that it is important to take into account the correct evolutionary context in experiments aimed at the identification of viral suppressors of RNAi. For example, we note that we would not have detected RNAi suppressive activity in DimmNV, if we had solely relied on experiments in *D. melanogaster*.

In striking contrast to DimmNV, DmelNV VP1 did not show species-specific activity. It can engage in an interaction with both Dimm and Dmel AGO2 and, accordingly, it inhibited slicer activity in both *D. immigrans* and *D. melanogaster* embryo lysates. We suggest that there are two potential explanations for this. First, it may be that these viruses differ in natural host range; the broader-spectrum functionality of DmelNV VP1 across divergent hosts could be maintained by selection if DmelNV has a wider host range than DimmNV. In support of this hypothesis, although none of these three viruses was identified from the other host species, DmelNV (but not DimmNV) has been identified in wild *Drosophila simulans* (DJO, unpublished data). Second, if there is not a substantial trade-off associated with host-specialization and if DmelNV has colonized *D. melanogaster* quite recently, it could just be a matter of time until DmelNV loses its broad-spectrum VSR.

We successfully reconstituted Dimm AGO2-based silencing in *D. melanogaster* cells. This result suggests that the limited amino acid identity with Dmel AGO2 (∼63%) does not impede its ability to interact with Dmel Dicer-2 and R2D2 or other components of RISC and RISC-loading complexes. Thus, even though RNAi genes are rapidly evolving and show high rates of adaptive substitution, these results imply that this diversification has not impeded cross-species interactions of RNAi genes, even over the tens of millions of years that separate *D. melanogaster* and *D. immigrans*. This conservation of function may imply that the need for interaction between Dicer-2, R2D2, AGO2, and other RNAi pathway genes imposes a constraint on the evolution of these genes, and thus their opportunity to evolve in response to virus-mediated selection.

Together, our results suggest that rapid co-evolution between RNA viruses and their hosts may result in host species-specific activities of RNAi suppressor proteins. Moreover, our observation that DimmNV VP1 enhances viral RNA levels in a host-specific manner, suggest that viral RNAi suppressors are putative host-specificity factors.

## Materials and Methods

### Identification and sequencing of novel Nora-like viruses

Wild *Drosophila* populations were surveyed for the prevalence of Dmel Nora virus using RT-PCR (unpublished data; PCR primers: forward 5′-GACCATTGGCACAAATCACCATTTG-3′, reverse 5′-TCTTAGGCCGGTTGTCTTCACCC-3′), which resulted in the identification of Nora virus-like PCR products from *D. immigrans* and from members of the obscura group (sampled in Edinburgh, UK; longitude 55.928N, latitude 3.170W). A metagenomic approach was then used to obtain near-complete viral genomes. Flies were collected from elsewhere in the UK and samples were pooled by species for RNA extraction and Illumina double-stranded nuclease normalized RNA-sequencing. For *D. subobscura*, only male flies were used as females are difficult to distinguish morphologically from close relatives. RNA was extracted from each collection using a standard Trizol (Invitrogen) procedure, according to the manufacturer's instructions, and pooled in proportion to the number of contributing flies. In total, the two pools comprised 338 male *D. subobscura* (60 flies collected July 2011 Edinburgh 55.928N, 3.170W; 60 flies October 2011 Edinburgh 55.928N, 3.170W; 38 flies July 2011 Sussex 51.100N, 0.164E; 180 flies August 2011 Perthshire 56.316N, 3.790W) and 498 *D. immigrans* (63 flies, July 2011 Edinburgh 55.928N, 3.170W; 285 flies July 2011 Edinburgh N55.921, W3.193; 150 flies July 2011 Sussex 51.100N, 0.164E). Total RNA was provided to the Beijing Genomics Institute (Hong Kong) for normalization and 90-nt paired-end Illumina sequencing. Paired-end reads were quality trimmed using ConDeTri version 2 [76] and assembled *de novo* using the Trinity transcriptome assembler with default settings (r2011-08-20, ref. [77]). We used tBlastn with a DmelNV protein query to identify two partially overlapping Nora-like contigs from *D. immigrans*, and a single contig from *D. subobscura*. Quality-trimmed paired-end reads were mapped back to these contigs using Stampy (version 1.0.21, ref. [78]) to obtain a consensus sequence, based on majority calls at each position. In total, 286,242 reads mapped to DimmNV (0.45% of all reads derived from *D. immigrans*, median read depth 1200-fold) and 68,914 reads mapped to DsubNV (0.13% of all reads derived from *D. subobscura*, median read depth 133-fold). Consensus sequences have been submitted to GenBank under accession numbers KF242510 (DsubNV) and KF242511 (DimmNV).

### Tree inference and sequence analysis

The relationship between DmelNV (GenBank NC_007919.3; [57]), DsubNV, DimmNV and other Nora-like sequences was inferred from VP4 (capsid protein), which is the most conserved gene and the one with the most coverage in the non-Drosophila sequences. The other Nora-like sequences included *Nasonia vitripennis* Nora-like virus (GenBank FJ790488; [60]), *Haematobia irritans* Nora-like virus (GenBank HO004689, HO000459, and HO000794; [59]), and two Nora-like sequences newly identified here in the transcriptomes of *Spodoptera exigua* (GenBank GAOR01000957; [79]) and *Chrysopa pallens* (GenBank GAGF01018485; [80]). We excluded sequences virtually identical to DmelNV that appear in the transcriptomes of *Leptopilina boulardi* and *Leptopilina heterotoma* (GenBank GAJA01006738, GAJC01010128 and GAJA01017939; [81]), as these species are widely cultured on *D. melanogaster* in the laboratory. For protein alignment, see text S1.

For the *N. vitripennis* Nora-like virus we selected the longest sequence (FJ790488) for analysis. Two approaches to phylogenetic inference were used. First, MrBayes (v3.2.1, ref. [82]) with discrete gamma-distributed rate variation and model-jumping between amino acid substitution models. Two parallel runs of four heated chains were used, and convergence was assessed by examination of the potential scale reduction factor (PSRF) and the variance in split-frequencies between runs (PSRF ∼1 for all parameters; variance in split-frequencies <0.001). Second, a maximum-likelihood analysis was run using PhyML [83] under a WAG amino-acid substitution model [84] with discrete gamma-distributed rate variation. Data were bootstrapped 1000 times to infer bootstrap node-support. The nonsynonymous divergence along each of the branches leading to DmelNV, DsubNV, and DimmNV was inferred using the method of Li [85], relative to an ancestral sequence inferred by maximum likelihood using PAML [61]. Sliding windows of 50 codons wide were placed every 30 codons. Nominal genome-wide ‘significance’ thresholds for peaks were derived by repeating the sliding-window analysis on 1000 randomizations of codon-position order.

### Cloning

The following constructs were described previously: all DmelNV VP1 constructs [14], pAFW-AGO1 and pAFW-AGO2 [86], pAFW (Drosophila Genomics Resource Center, https://dgrc.cgb.indiana.edu), pMT-Luc [38], pMT-Rluc [38], pRmHa-*Renilla*-hairpin [87], pAc5-V5-His-A (Invitrogen), and pAc5-V5-His-Ntag [14].

cDNA of *D. immigrans* and *D. subobscura* was made using Promega MMLV-RT in the presence of Promega RNasin Plus according to manufacturer's instructions. Subsequently, DimmNV VP1 and DsubNV VP1 sequences were PCR amplified from *D. immigrans* and *D. subobscura* cDNA and cloned as full-length and deletion constructs downstream of the V5-His tag in pAc5-V5-His-Ntag (details available upon request).

The *D. immigrans* AGO2 cDNA sequence (GenBank KF362118), including partial 5′ and 3′ UTRs, was PCR amplified using the primer pair 5′-TGCAGCAAAAATTAGAAGCAAA-3′ and 5′-AGCCGTACCTAGAACCAGCA-3′. The resulting PCR product was used as a template in a nested PCR using primer pair 5′-AGTTCTAGACCGCGGGAATGGGTAAAAAGAACAAGTTCAAACCA-3′ and 5′-AGTTCTAGACCGCGGGAAGCGCTGTGGCACAGCTTCCGC-3′. The nested PCR product was subsequently cloned into the pAFW vector using the SacII and SalI restriction sites. To fuse the DimmNV VP1^ΔN295^ protein to the C-terminus of the maltose binding protein (MBP), we PCR amplified the VP1 coding sequence from pAc5.1-Ntag-DimmNV VP1^FL^ with primer pair 5′-AGTGGATCCCCAAAACTTCCAAGTGTACCTTCAAAG -3′ and 5′-GGTGTCGACTTAGTTTTGTTTATTTTTGTACCAATCGTTGG -3′. The DsubNV VP1^ΔN281^ sequence was amplified from pAc5.1-Ntag-DsubNV VP1^FL^ with primer pair 5′-TGACGGATCCCCAAACAAACCTCTAAAACC -3′ and 5′-ACTGGTCGACTCATTGTTGCTGAGTTGATTTG -3′. The resulting PCR products were cloned into the pMal-C2X vector (New England Biolabs) using BamHI and SalI restriction sites.

### RNA silencing reporter assays

Double-stranded RNA was generated by *in vitro* transcription using T7 promoter-flanked PCR fragments as a template, as described previously [88]. For production of AGO2 dsRNA, a fragment of the coding sequence or the 3′ untranslated region of Dmel AGO2 was PCR amplified using primer combination 5′-TAATACGACTCACTATAGGGAGATACTATGGTGAAGAACGGGTCG-3′ and 5′-TAATACGACTCACTATAGGGAGAGAACATGTCCTCAATCTCCTCC-3′, or primer combination 5′-TAATACGACTCACTATAGGGAGAGCAACGTATTGAATCTTATT-3′ and 5′-TAATACGACTCACTATAGGGAGAAGAACAATATTTGGCGGACC-3′, respectively.

miRNA and RNAi sensor assays in *Drosophila* S2 cells were performed as described [14], [88]. For hairpin-induced silencing of the Rluc reporter, 5×10^4^ S2 cells were seeded per well in a 96-well plate. The seeded cells were co-transfected with 10 ng pMT-Fluc, 10 ng pMT-Rluc, 50 ng pRmHa-*Renilla*-hairpin, and 50 ng of expression plasmids encoding VP1 and/or AGO per well using Effectene transfection reagent (Qiagen). The pAc5-Ntag-DmelNV VP1^Δ284^ and pAc5-Ntag-DimmNV VP1^ΔN295^ plasmids were used for VP1 expression. For knockdown of endogenous AGO2, 5 ng of AGO2 dsRNA or control dsRNA was co-transfected along with reporter plasmids. Two days after transfection, the expression of the luciferase reporters and the Rluc hairpin was induced by the addition of 0.5 mM CuSO_4_ per well. The next day, cells were lysed and Fluc and Rluc activity was measured with the Dual luciferase reporter assay system (Promega) according to manufacturer's protocol.

### Immunoprecipitation and western blotting

For immunoprecipitations, S2 cells were seeded in 6-well plates at a density of 2×10^6^ cells per well. The next day, cells were transfected with AGO2 and/or VP1 expression plasmids using Effectene transfection reagent (Qiagen). Expression plasmids encoding DmelNV VP1^ΔN351^, DmelNV VP1^ΔN284^, or DimmNV VP1^ΔN295^ were used for co-immunoprecipitation experiments, as indicated in the figure legends. Three days after transfection, cells were washed twice with PBS and subsequently resuspended in lysis buffer (30 mM HEPES-KOH, 150 mM NaCl, 2 mM Mg(OAc), 0.1% NP-40, 5 mM DTT) supplemented with protease inhibitor cocktail (Roche). After incubation on ice for 10 minutes, the samples were passed forty times through a 25-gauge needle, followed by incubation on ice for 10 minutes. Subsequently, cell lysates were centrifuged at 13,000 rpm for 30 minutes and a sample of the supernatant was taken to analyze the input for IP. To remove proteins that non-specifically bind to the IP beads, the remaining supernatant was incubated with Pierce protein G agarose at 4°C for 5 hours while mixing end-over-end. Next, the protein G agarose was separated from the supernatant by centrifugation, after which the supernatant was incubated overnight with anti-V5 agarose affinity gel (Invitrogen) at 4°C while mixing end-over-end. The next day, the anti-V5 agarose was separated from the supernatant by centrifugation, and a sample was taken from the supernatant. After the remaining supernatant was removed, the V5-agarose was washed three times with lysis buffer, and three times with either wash buffer 150 (25 mM Tris-Cl, 150 mM NaCl) or wash buffer 200 (25 mM Tris-Cl, 200 mM NaCl). All wash steps were done with 40 to 60 times beads-volume of wash buffer. Subsequently, the beads were boiled in SDS sample buffer at 95°C for 10 minutes, followed by a brief centrifugation step to collect the beads at the bottom of the tube. The proteins in the supernatant were then separated on a SDS-PAGE gel, after which they were transferred onto a nitrocellulose membrane by western blot. Primary antibodies used for western blot detection were anti-FLAG-M2 (1∶1000 dilution; Sigma), anti-V5 (1∶5000 dilution; Invitrogen), anti-AGO2 (1∶500 dilution; generously provided by the Siomi lab), and anti-tubulin-alpha (1∶1000 dilution, Sanbio); secondary antibodies were goat anti-mouse-IRdye680 (1∶15,000 dilution; LI-COR), and goat anti-rabbit-IRdye800 (1∶15,000 dilution; LI-COR). All western blots were scanned using an Odyssey infrared imager (LI-COR biosciences).

### Purification of recombinant protein

To purify recombinant VP1 as MBP fusion proteins, the pMal-C2X-DimmNV VP1^ΔN295^ and the pMal-C2X-DsubNV VP1^ΔN281^ plasmids were transformed into the *Escherichia coli* BL21 (DE3) strain. Subsequently, expression of recombinant protein was induced by addition of 0.2 mM IPTG. Protein expression was allowed to proceed overnight at 18°C. The next day, recombinant MBP-DimmNV VP1 and MBP-DsubNV VP1 were purified using amylose resin (New England Biolabs) according to the manufacturer's protocol. Purified protein was subsequently transferred to a dialysis membrane (molecular weight cut-off 12–14 kDa) and incubated overnight in dialysis buffer (20 mM Tris-Cl, 0.5 mM EDTA, 5 mM MgCl_2_, 1 mM DTT, 140 mM NaCl, 2.7 mM KCl) at 4°C, followed by a second dialysis step for 5 hours at 4°C. The dialyzed protein solution was stored at −80°C in dialysis buffer containing 30% glycerol. Purification of MBP-DmelNV VP1^ΔN284^ has been described previously [14].

### Slicer assays

A new *D. immigrans* isofemale line was established from flies collected in June 2012 in Edinburgh (Coordinates 55.921N, 3.193W). *D. immigrans* was cultured similarly as *D. melanogaster* on standard media. Embryo lysates were generated from *D. immigrans* and from an RNAi-competent *D. melanogaster* laboratory control strain (*w* ^1118^). *In vitro* target RNA cleavage assays in *D. melanogaster* embryo lysates were performed as described [14]. Minor changes were incorporated for the slicer assay in *D. immigrans* embryo lysate: the reaction contained 0.9 mM MgCl_2_ and was allowed to proceed for 5 hours at 25°C before RNA extraction. Suppressor activities of MBP-DmelNV VP1^ΔN284^, DsubNV VP1^ΔN281^, and MBP-DimmNV VP1^ΔN295^ proteins were analyzed in slicer assays.

### Virus infections

To produce recombinant Sindbis viruses, N-terminal V5 tagged DmelNV VP1^ΔN284^ and DimmNV VP1^ΔN295^ were PCR amplified from the respective insect expression vectors using primers V5 Fw: AGTTCTAGAAACATGGGTAAGCCTATCC; Dmel VP1 Rv: GGTTCTAGATTAACATTGTTGTTTCTGCGAG; and Dimm VP1 Rv: TGACTCTAGATTAGTTTTGTTTATTTTTGTACC. PCR products were cloned into the XbaI site following the second subgenomic promoter of the pTE3'2J vector [89]. The resulting plasmids were linearized with XhoI, and in vitro transcribed using the mMESSAGE mMACHINE SP6 High Yield Capped RNA Transcription kit (Ambion). Transcribed RNA was then purified using the RNeasy kit (Qiagen) and transfected into BHK-21 cells to produce infectious virus. Supernatant was harvested and titered by plaque assay on BHK-21 cells. Sindbis-GFP was described previously [69].

The replicative capacity of recombinant viruses was analyzed on *Dicer-2* deficient C6/36 cells. The cells were cultures as described previously [90] and inoculated at an multiplicity of infection of 0.01. Cells were harvested directly after inoculation (t = 0) and at 24 h thereafter and total RNA was isolated using isol-RNA lysis reagent (5 Prime). The RNA was treated with DNaseI and used as template for cDNA synthesis using Taqman reverse transcription reagents (Roche). Viral RNA levels were determined by qPCR using the GoTaq qPCR Master Mix (Promega) and primers for either Sindbis (SINV NS4 Fw: AACTCTGCCACAGATCAGCC; SINV NS4 Rv: GGGGCAGAAGGTTGCAGTAT) and Aedes Albopictus RpL5 for normalization (Aalb RpL5 Fw TCGCTTACGCCCGCATTGAGGGTGAT; Aalb RpL5 Rv: TCGCCGGTCACATCGGTACAGCCA).

### Fly infections and viral RNA quantification

Flies (*Drosophila melanogaster w* ^1118^ and *Drosophila immigrans*) were grown on standard yeast/agar medium at 25°C on a 12-h light/dark cycle. Flies were cured of *Wolbachia sp*. by tetracycline treatments as described in [91]. Five to seven-day-old female flies were CO_2_-anesthetized and intrathoracical single injections of 50.6 nL, corresponding to 5,000 plaque forming units for each virus, were performed using a nanoinjector Nanoject II (Drummond Scientific Company) as described in [92].

For each time point, total RNA from three independent pools of three flies was isolated using TRIzol Reagent (Life Technologies). RNase-free DNase I treatment (Roche) was performed according to manufacturer's instructions, followed by acid-phenol/chloroform (Life Technologies) inactivation. Total RNA was quantified using a ND-1000 NanoDrop spectrophotometer (Thermo Fisher Scientific). Reverse transcription was performed using SuperScript II Reverse Transcriptase with random hexamers as primers (Life Technologies) on 2 µg of total RNA. Quantitative PCR was performed with three technical replicates for each cDNA sample using FastStart SYBR Green Master (Rox) (Roche) on a ViiA7 Real-Time PCR instrument (Life Technologies). As negative controls, cDNA reactions without reverse transcriptase and PCR amplification without cDNA template were included. Oligonucleotide primers were as follows (F, forward; R, reverse) Sindbis virus: SINV-NSP3_F, AAAACGCCTACCATGCAGTG; SINV-NSP3_R, TTTTCCGGCTGCGTAAATGC, and for normalization *Dimm*-AGO2_F, TTTTGTGCTGGGCGACAAAC; *Dimm*-AGO2_R, ATTCACCGCTTCGCAAATCG and *Dmel*-RpL32_F, CGGATCGATATGCTAAGCTGT; *Dmel*-RpL32_R, GCGCTTGTTCGATCCGTA. Relative viral RNA levels were calculated using the 2^−ΔΔCT^ method [93] relative to input viral RNA, determined in flies that were harvested immediately after inoculation. Following log-transformation to homogenize variances, a T-test was used to compare relative RNA levels in SINV-VP1 recombinants to those in SINV-GFP.

### GenBank accession numbers

*D. immigrans* AGO2 cDNA sequence: KF362118; DsubNV consensus sequence: KF242510; DimmNV consensus sequence: KF242511; DmelNV: NC_007919.3; *Nasonia vitripennis* Nora-like virus: FJ790488; *Haematobia irritans* Nora-like virus: HO004689, HO000459, and HO000794; Transcriptome of *Spodoptera exigua*: GAOR01000957; Transcriptome of *Chrysopa pallens*: GAGF01018485.

## Supporting Information

Figure S1 **Sequence alignment of Nora viruses from different** ***Drosophila*** **species.** (**A**) The 5′ terminal sequence of *D. immigrans* Nora-like virus (DimmNV) and *D. subobscura* Nora-like virus (DsubNV) obtained by metagenomic RNA-sequencing was aligned to the first 70 nt of the 5′ UTR of *D. melanogaster* Nora virus (DmelNV, GenBank NC_007919.3). The DmelNV 5′ sequence had been determined by 5′RACE [57], suggesting that RNA-sequencing recovered near-complete sequences of DimmNV and DsubNV. (**B**) VP1 sequences of DmelNV, DsubNV, and DimmNV were aligned with Clustal Omega using default settings. Arrows indicate the first amino acid of the N-terminal deletion mutants (ΔN) and the last amino acid of the C-terminal deletion mutants (ΔC) that were used in this study.
(TIF)

Figure S2 **DsubNV VP1 and DimmNV VP1 do not suppress RNAi in** ***D. melanogaster*** **.** (**A**) Western blot analysis of V5-tagged full-length (FL) or N-terminal deletion (ΔN) constructs of DsubNV VP1 or DmelNV VP1. VP1 proteins were detected with anti-V5 (α-V5) antibody. Tubulin (α-tub) was used as a loading control. (**B**) dsRNA-induced RNAi sensor assay in *D. melanogaster* S2 cells. Firefly luciferase (Fluc) and Renilla luciferase (Rluc) reporter plasmids were co-transfected with plasmids encoding DmelNV VP1, DsubNV VP1, or a control plasmid (Vector). Two days after transfection, cells were soaked in medium containing Fluc dsRNA or control dsRNA. One day later, luciferase activities were measured and Fluc counts were normalized to Rluc counts and expressed as fold silencing relative to the corresponding control dsRNA treatment. (**C**) siRNA-induced RNAi sensor assay in S2 cells. The assay was done as described in panel B, except that siRNAs targeting Fluc (Fluc siRNA) or control siRNAs (Ctrl siRNA) were co-transfected with the plasmids instead of soaking the cells in dsRNA. Bars represent means and standard deviations of three independent biological replicates. One-way ANOVA followed by Dunnett's *post hoc* test was used to evaluate whether VP1 constructs significantly suppressed RNAi relative to the vector control (light gray bar). *** *P*<0.001; ns, not significant. (**D**) *In vitro* RNA cleavage (slicer) assays in lysates from *D. melanogaster* embryos. Radioactively cap-labelled target RNA was incubated in embryo lysate together with a non-specific control siRNA (lane 1) or a target specific siRNA (lanes 2–5). Target cleavage was determined either in the absence of recombinant protein (lane 2) or in the presence of 0.3 µM of MBP (lane 3), MBP-DmelNV VP1 (lane 4), or DsubNV VP1 (lane 5).
(TIF)

Text S1 **Amino acid sequence alignment used for** **Figure 1B** **.**
(DOCX)
