# Supplementary figures and images for: Novel Drosophila Viruses Encode Host-Specific Suppressors of RNAi
Source: PLoS Pathog. 2014 Jul 17;10(7):e1004256. doi: 10.1371/journal.ppat.1004256 (PMC4102588; doi:10.1371/journal.ppat.1004256)

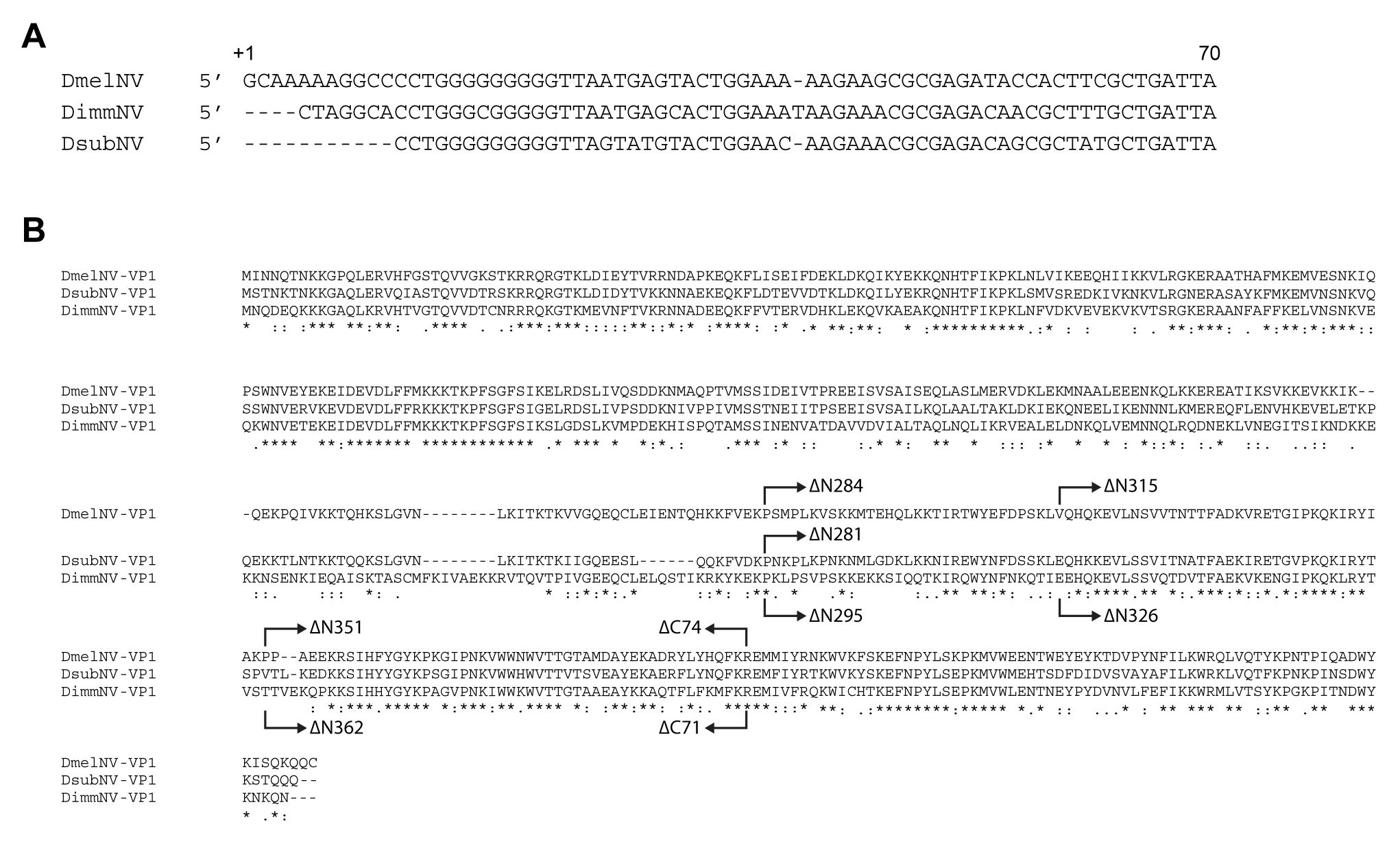

Supplement: Figure S1 — Sequence alignment of Nora viruses from different Drosophila species. (A) The 5′ terminal sequence of D. immigrans Nora-like virus (DimmNV) and D. subobscura Nora-like virus (DsubNV) obtained by metagenomic RNA-sequencing was aligned to the first 70 nt of the 5′ UTR of D. melanogaster Nora virus (DmelNV, GenBank NC_007919.3). The DmelNV 5′ sequence had been determined by 5′RACE [57], suggesting that RNA-sequencing recovered near-complete sequences of DimmNV and DsubNV. (B) VP1 sequences of DmelNV, DsubNV, and DimmNV were aligned with Clustal Omega using default settings. Arrows indicate the first amino acid of the N-terminal deletion mutants (ΔN) and the last amino acid of the C-terminal deletion mutants (ΔC) that were used in this study. (TIF) [file ppat.1004256.s001.tif]

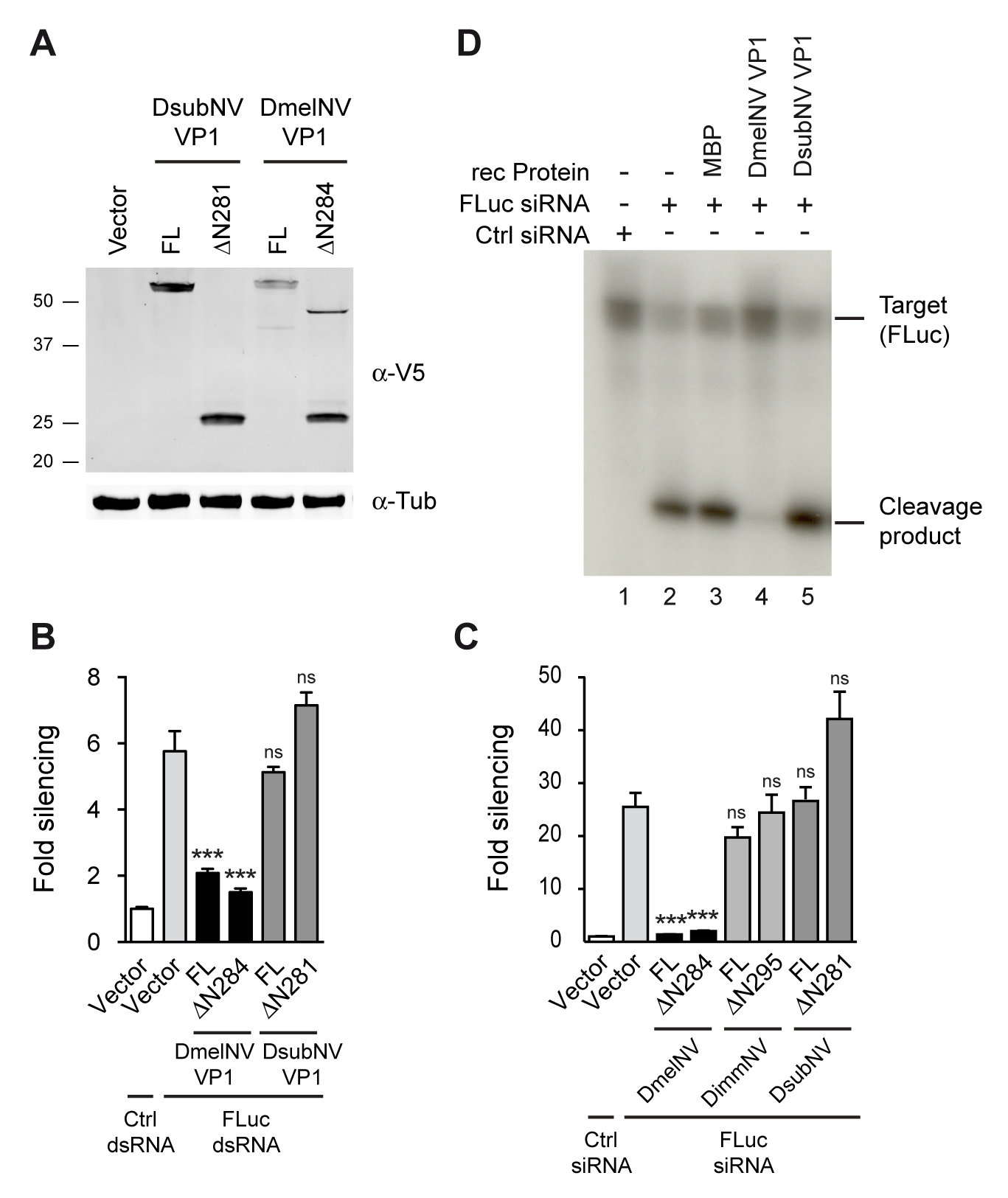

Supplement: Figure S2 — DsubNV VP1 and DimmNV VP1 do not suppress RNAi in D. melanogaster . (A) Western blot analysis of V5-tagged full-length (FL) or N-terminal deletion (ΔN) constructs of DsubNV VP1 or DmelNV VP1. VP1 proteins were detected with anti-V5 (α-V5) antibody. Tubulin (α-tub) was used as a loading control. (B) dsRNA-induced RNAi sensor assay in D. melanogaster S2 cells. Firefly luciferase (Fluc) and Renilla luciferase (Rluc) reporter plasmids were co-transfected with plasmids encoding DmelNV VP1, DsubNV VP1, or a control plasmid (Vector). Two days after transfection, cells were soaked in medium containing Fluc dsRNA or control dsRNA. One day later, luciferase activities were measured and Fluc counts were normalized to Rluc counts and expressed as fold silencing relative to the corresponding control dsRNA treatment. (C) siRNA-induced RNAi sensor assay in S2 cells. The assay was done as described in panel B, except that siRNAs targeting Fluc (Fluc siRNA) or control siRNAs (Ctrl siRNA) were co-transfected with the plasmids instead of soaking the cells in dsRNA. Bars represent means and standard deviations of three independent biological replicates. One-way ANOVA followed by Dunnett's post hoc test was used to evaluate whether VP1 constructs significantly suppressed RNAi relative to the vector control (light gray bar). *** P<0.001; ns, not significant. (D) In vitro RNA cleavage (slicer) assays in lysates from D. melanogaster embryos. Radioactively cap-labelled target RNA was incubated in embryo lysate together with a non-specific control siRNA (lane 1) or a target specific siRNA (lanes 2–5). Target cleavage was determined either in the absence of recombinant protein (lane 2) or in the presence of 0.3 µM of MBP (lane 3), MBP-DmelNV VP1 (lane 4), or DsubNV VP1 (lane 5). (TIF) [file ppat.1004256.s002.tif]
