# Supplementary material for: Novel Drosophila Viruses Encode Host-Specific Suppressors of RNAi
Source: PLoS Pathog. 2014 Jul 17;10(7):e1004256. doi: 10.1371/journal.ppat.1004256 (PMC4102588; doi:10.1371/journal.ppat.1004256)
Supplement: Text S1 — Amino acid sequence alignment used for Figure 1B . (DOCX) [file ppat.1004256.s003.docx]

>DsubNora

--------------------MQNPTQTMHIYDMPLRVIAGLSTLAKT---PDEDDNTSTG

VVVSEVGEPQATTHPAWIDSFISYQLRAPRETM-NPDFIFGGADVGNAFSAFLPRRFSAP

AIGTRLVIDPVCTYPQYVMMQLYN---FFHADFYYIIHVPAPLGTGIYLKVYAPELDTST

VTRGIRFKPSALPTVAVSLPWSNDLSNVP----VSEGRLGQSGGTIVIETIEDNSNETVN

TPLSITVW---------CCMANIRMTG-YAIADSSAYNHPGMNFMPLPTPTPPPA-----

-----QIVGEEQADNEISAEGGK-PVQELIYDQSAIPVAPTVEKEAEQPETP-AANIATR

KNDTGHLATKWYDFVKIKLSNPANMSWTVLTIDPYNNVTLSRDGESMVLPWRRNVWTTGS

KAIGYIRTMVAQINIPRPPQISGVLEVK--DSIN-NSSISLIEFGGKVEVPLIPKVLNGL

ASSTKLPRHWLNPWMRTAESKVELAYRIIAFNRTSDIADLNVSVLLRPGDSQFQLPMKPD

NNVDTRHIELVDMLMHEYNQ---LRIDG-----EEQSLPEESVPH---------------

-----------------------TMNPAQFITPAVAQTAEQYNVHTAIGENEELELDEFP

VLVFKGEIPVGEVSSIQLDLAKIYDFAWDGEQNAISQKFQRFAHLIPKSAGGFGPIIGNY

TITCNLPTGVAGRIVHNCLPGDCVDLAVSRIFGLK-SLLGIAGSAVSAIGGPLMNGLVNT

AAPILSGAA---------HAIGGNVVGGLADGVINTVSSLLTPKEKEQPSANSNAISGDI

PISRFVEMLKYVQTNYQDNPVFPTLLIEPQN-FISNAMSALTKIPIEVFANMRNVDVERN

LFDRAIVPVVEEETLSDVVIPSHSYAYIMRDFLQSKQAFRPGTKQNVYFKQFLTVLQTR-

-NTKSHINLKDIITCPIDDESVALKIGRVKHYLSKNLSGETTEEFSRTDTGSEVNKIRKI

LINRTS------TNET*-------

>DimmNora

--------------------MQNPTQTMHVYDMPLRVIAGLSTLAKS---QEEDENTSTG

VVVSEVGEPQVVNHPAWIDPFVAYQLRAPRENL-TPDFIFGRATVGNAFSAFLPRRFSAP

AVGTRLVIDPVCTFPQYVMLQLYN---FYHADFFYIVHVPAPLGTGIYLKVYAPELDSTT

VTRGIRFKPSASPTMAFSVPWSNDLSSVP----TTSGRLGQSGGSIVIETIEDNSNETVN

TPLSITVW---------CCMANVDMTG-YAIADATAYNYPGMNFIPTPVPTEAIAPANPD

DKTPIPIYGEEQADNEVTAEGGK-LVQELIYDHSAIPVAPQVEKEAEQPEAP-ASTVATR

KNDTGHLATKWYDFVKISLSNPKDMSWTTLTIDPYNNVTLSRNGEAMVLPWRRNVWTTGS

KAIGYIRTMAAQINIPRPPQISGVLEVK--DSIN-NSSISLVEFGGKVEIPLLPKVFNGL

ANSTKLPRHWLNPWMRTAESKIELQYRIIAFNRTSDIADLSVSVLLRPGDSTFQMTTKPD

NSVDTRHFELVDRLMYEFEN---LRIHG-----EEQGLPEETPER---------------

-----------------------EVNPAQFITPAVGFTAEKYNLHEELGECEDLELDEFP

VLVFKGEVPVGEVTPIQLDLATIYDFAWDGEQNAISQKFQRFAHLIPKSAGGFGPVIGNY

TITANLPTGVAGRILHNCIPGDCVDLSVSRIFGLK-SLFGLMGTAVTSIGGPLLSGVVNT

AAPILSGAA---------HAIGGNVVGGLTDTVLGAASNLLTHKEKEQPSANAEAMAGDI

PISRFVEMLKYVKGNYESNPVFPTLLVEPQN-FVSNALAALTKIPIEVFANMRNIKVERN

LFDRTVTPIVEEEAIADIVIPNHAYAFILRDFLQCKRAFKPGTKQQVYFKQFLTVLSQR-

-NTRTHITLKDITSCSIDEESVATKIERVKHYLDSNLGGETTEEFSRTDTGLPLNAIRKL

VLSESKRRTERYVAETVFPSVRQ*

>DmelNora

--------------------MQNPTQTMHIYDMPLRVIAGLSTLAKT---TEEDDNTSTG

IVVSEVGEPQVVNHPAWIDPFVAYQLRAPRKNI-TPDFIFGRADIGNAFSAFLPRRFSAP

AVGTRLVVDPVFTYQQRTVLGLYN---YFHADFYYIVHVPAPLGTGIYLKIYAPEFDTTT

VTRGIRFKPSASPTIALSVPWSNDLSTVE----TSVGRVGQSGGSIVIETIEDNSNETVN

TPLSITVW---------CCMANIKATG-YRHADTSAYNEKGMNFIPVPVPKPPVPPTKP-

------ITGEEQADNEVTAEGGK-LVQELVYDHSAIPVAPVVETQAEQPEVP-VSLVATR

KNDTGHLATKWYDFAKISLSNPANMNWTTLTIDPYNNVTLSRDGESMVLPWRRNVWTTGS

KSIGYIRTMVAQINIPRPPQISGVLEVK--DSIN-NSSISLVEFGGKVEIPIIPKVMNGL

ATTASLPRHRLNPWMRTAESKVELQYRIIAFNRTSDIADLNVSVLLRPGDSQFQLPMKPD

NNVDTRHFELVEALMYHYDS---LRIRG-----EEQSLPENAPNA---------------

-----------------------VSNPQQFITPATALSAEEYNVHEALGETEELELDEFP

VLVFKGNVPVDSVTSIPLDLATIYDFAWDGEQNAISQKFQRFAHLIPKSAGGFGPVIGNY

TITANLPTGVAGRILHNCLPGDCVDLAVSRIFGLK-SLLGVAGTAVSAIGGPLLNGLVNT

AAPILSGAA---------HAIGGNVVGGLADAVIDIGSNLLTPKEKEQPSANSSAISGDI

PISRFVEMLKYVKENYQDNPVFPTLLVEPQN-FISNAMTALKTIPIEVFANMRNVKVERN

LFDRTVVPTVKEATLADIVIPNHMYGYILRDFLQNKRAFQSGTKQNVYFQQFLTVLSQR-

-NIRTHITLNDITSCSIDSESIANKIERVKHYLSTNSSGETTEEFSRTDTGLLPITTRKI

VLGESKRRTERYVAETVFPSVRQ*

>H.irritans_Nora

--------------------MTNPTMNISVYDMPLRVIAGLSTLAKT---PEEDDSIETG

MVVSEVGEPHVTSHPAWCDEYVSYHLRAPRQLR-NPDFIFGGMTLGNAFSAYLPRRFAAP

AVGTRLVINPVVTKAQQTLINMYN---YIHANVHYIIHVPAPLGTGIYLXXXXXXXXXXX

XXXXXXXXXXXXXXXXXXXXXXXXXXXXX----XXXXXXXXXXXXXXXXXXXXXXXXXXX

XXXXXXXXXXXXXLRSGCCVADIELTG-YAIADTQGYDIPGLNFNPVIS-----------

-----LVTGEEQADNEVSAEGGK-IVDELSYDKSAIPVAPVVEQTAAAPDVP-AETTATR

KNDTGHLATKWYDFVKINLSAPTDLSWTVLTIDPYNDIRLSKDGEALVLPWKRNVWTTGD

KDIGYIRTLAAQINVPRPPQISGVLQVK--DSIN-NSSISLIEFGGKVKIPLLPEVFNGL

KTPKTLTRHWLNPWMRTAESKIQLAYRIIAFNRTSDIADLSVTVLLRPGGSQFQLPLKPE

RRIDSRHIDIVNMLMDEFNR---FAIRG-----EEQSLPEESPAN---------------

-----------------------LPSTNQFITPMTAQTAEQYNLHSDLGDEEDLELDEFP

VLVFRGEIPTDEVTSIQLDLAKIYDFAWDGDENAISQKFLRFAHIIPKSAGGFGPVIGNY

TITANLPTGVAGRIVHNCLPGDCVDLSVSRIFGLK-SLLGIAGSAVSAIGGPLMNGLVNT

AAPILSGAAX--------HAIGGDVVGGLADKVLDIASNVLTPKEKAQPSANSNSISGDI

PISRFVEFLKYVKTNYESDPIFPTLLIEPQN-FLTNAMTKLERIPIEVFANMRNVKVERN

LFDRTVVPEVVEEQISDLVIPNEKLGYVIRDFLQSKHAFRPGTKQHTYFKQFLTTLLNR-

-KSNRYVTLRQIVNTPVDDESISEQLERVKRYLRQTLDGETPDEFS----------ARKL

LL----------------------

>FJ790488_Nasonia_vitripennis

---------------------MANTTVIKVHQRPIQSPELPGVLPRNQAIVELKTAEEQG

ALISEVGHP--TPFKIADVDLMAFTLRKARKSK-YPSHIYGNLEFGDAFSFWSPRVIDIP

AEGSSIQIDPEWSTFHKSVLDFYA---CRSASAFYVIHVPLPLGASLYLEATCPEQTLET

VTRGVRWRPNVLPSASFFVGWDYPKKWKLG---YQKLQGGYTGLSLKLRTIQSSNSSSSQ

VPLKALVF---------CCMTDVRGMGLRGITTSDPD---AFKFQPVASPRTWYTEMDS-

--------GETFVAQPGAIGDTPTAAVPETPGTKDTPVTAKTEEIVKVQSTP-TQAVSSS

KPNVKAVSQKFIFIERIKVSTADMGKPIVYQFKPSS---RTVKGDDLGLPYRRNVWCTGV

MRDGFNRECEFKITSTRSPQIAGIVQVRVIKSSDLGYSCRYHELGGQSTTVMAPPVVHE-

-PSSLTERTVSTGWHKASQATYTLILNVLCINRTADSKEATLSIYARTPNVHFSVPTKPK

KTPKPPPPPKPDKPLEDGTKDSVLQIAHLLQTIVPYGXXXXXXXX---------------

-----------------------XXXXXDIIAPPSVDNGVEYGFSHPEGEIDYIDQDDFW

VCIDQFDLKPGQKHATPYCPWALSDILNPESESPIVQKMERFSNLEPRVGGDKGPAFGQY

RLIAHLPQNIAANIAHISVPGDMGVETVGLRFDILGSLLGIAGSALTSIGGPLITGAIDA

VQGIVKDVPIIGGVLNDLGDIAKEVVGGTGNSPTEPVDQPQQPTVTP-AGPTSKEVRGRL

EGPRYLDYIKMFTDDVTVNDVIGQVVFELVDLIGADKIPCRLLAKVGDMASFT-----RS

VFNRTVFPSSQMSFIVG--LSPAEMGYLLEQAAE-----RPNSNSQLVVASLLSSLSNYG

GQPILYVNFSETAVKSSYNDLYKXTYHLCPRSLIDREPVLAAETSSFAGSFSRFRRLKKH

SFPV*-------------------

>GAGF01018485_Chrysopa pallens

MNSNKDKKKSSKPGLRQNNSQQLPQTAITVYNMPGQNRVFLPTVPRT---PEDDDSIESG

STVSEVGFSEVVRLKCLGHTIAAYNLRSRRKGRSLPMYVRGGCSMGETFSQMIPIIVSLP

TVNNVITIDPVLPRRMLDFVTASSGYEFIRAQVVWVVHIPSPLGTALILRAWAPELDATT

ETRGVRWKPQSNTAIAFKMDWSSDIPFVRNTQTLTAVRDGQSGLSLKIQCVEDNSTEAVN

TPLTATVW---------CCVYNVTMSGQRNFTEADRGALLALNFKPQATPX---------

----------TDTSGEIQAEGVSNLTVTAPANTDTTPLVPELNQAAPASKKPDSYAGKTK

KNQIGALNQRFQHFNKFTVGGAPTLTWTNININPYN---ITGKGEAFNLPFRRNVWTSGS

MSMGYLSTLQVQVHVARPPQVSGTIQFR--DGNNPLATMYCVDFGGRLDFPLVPNVIN--

--IPVRPRHWNSPWFRTDEAACSLSYRLIAFNRTADIADVTVDIYIRPGASVFNTPIKPK

PRAVSALSELAQAYHDYEEEE-YARVYGGTIEIEQHGLPEEFSDRSRKIHFDNWCADNYD

IHQGDVDLAEAEFTPVWDKMCDCLDGSEDHVAPPAIDSPEEGMVNYVEHQDDDIDQDDYT

IRVWEGELTVGQPIAIPLNLSVLRDVSTISDETTIGQKFERFAHIMPATGGNLGPEIGTY

TIHTRLPTNVAASIAHVCVPDDLSDEVAARIFGLA-KVLDIATSAISSIGGPLISGVVQT

APKLISTVLPG-----PLGSLASKVVGGVANGLLGGKP--PAPQDPPGESSTPAAVGGKI

PIARFLEFLKPVASNLIADPSFSNLLVELIDSFGDLASRATPTIPVSVYVRMT-GTTDRS

VFNRTIVPRDNIANLT--WIPRDRVSYLFDMFGNHPNTFVEGTHQNRCFKQLMTVARQR-

-TNVPSINLQEVLTTEVPEDLDRQIQS---LLLARNSTGLMRILMTAREEAPALGPD---

------------------------

>GAOR01000957_Spodoptera exigua

---------------------------------------------RT---PDDDDSIENG

STVSEVGFSETVRLKCLGHSIAAYNLRSRRKGRSLPMYVRGGCSMGETFSQMIPIIVSLP

TVNNTITIDPVLPRRMLDFVTTSSGYEFIRAQVVWVVHIPSPLGTALVLRAWAPELDATT

ETRGVRWKPQSNTAIAFKMDWSSDIPFVRNTQTLTAVRDGQSGLSLKIQCVEDNSTDAVN

TPLTATVW---------CCVYNVTMSGQRNFAESDRGALLALNFKPQATPX---------

----------TDTSGEIQAEGVSNLTVTAPANTDTAPLVPELNKPAPTSKKPDSHAGKTK

KNQIGALNQRFQHFNKFTVGGTPTLTWTNINIDPYN---ITGKGEAFNLPFRRNVWTSGS

MSMGYLTSLQVQVHVARPPQVSGTIQFR--DGNNPLATAYNVDFGGRIDFPLVPNVMN--

--IPIRPRHYNSPWFRTDEAACTLSYRLIAFNRTADIADVTVDVYIRPGASVFNTPIKPK

PRAVSALSGLAQAYHDYEEAE-YVKVYGGVAEIEHHGMPEEFSDRSRKLHFDNWCAENQD

IHQGDEDLAEAEFTPIWDKMCDELDGSDDHVAPPAVDSPEEGMINYIEHQDDDIDQDDYT

IRVWEGELTIGQPLAIPXNLSVLRDVSTISDETTIGQKFERFAHIMPATGGNLGPEIGTY

TIHARLPTNVAASIAHVCVPDDLTDEVAARIFGLA-KVLDIAGSAISSIGGPLIAGAVQT

APKLIKTVLPG-----PLGSLASKVVGGVANGLLGGKP--PAPQDPPGESSTPTAIGGKI

PIARFLEFLKPVATNLVADPSFSNLLVELIDSFGDLAARATPTIPISVYVRMT-GATDRS

VFNRTVVPRDNVANLT--WVPRDRVSYLFDMFGNNPNAFVEGTHQNRCFKQLMTVVRQR-

-TNVPSINMQEVLATEVPEDLSRQIQS---LLLARNSTGLMQILMTAREEAPALGPD---

------------------------
